# Metrics of early childhood growth in recent epidemiological research: A scoping review

**DOI:** 10.1371/journal.pone.0194565

**Published:** 2018-03-20

**Authors:** Michael Leung, Nandita Perumal, Elnathan Mesfin, Aditi Krishna, Seungmi Yang, William Johnson, Diego G. Bassani, Daniel E. Roth

**Affiliations:** 1 Research Institute and Centre for Global Child Health, Peter Gilgan Centre for Research and Learning, The Hospital for Sick Children, Toronto, Canada; 2 Dalla Lana School of Public Health, University of Toronto, Toronto, Canada; 3 London School of Hygiene & Tropical Medicine, London, United Kingdom; 4 Department of Epidemiology, Biostatistics and Occupational Health, McGill University, Montreal, Canada; 5 School of Sport, Exercise and Health Sciences, Loughborough University, Loughborough, Leicestershire, United Kingdom; 6 Department of Paediatrics, The Hospital for Sick Children and University of Toronto, Toronto, Canada; Public Library of Science, UNITED KINGDOM

## Abstract

Metrics to quantify child growth vary across studies of the developmental origins of health and disease. We conducted a scoping review of child growth studies in which length/height, weight or body mass index (BMI) was measured at ≥ 2 time points. From a 10% random sample of eligible studies published between Jan 2010-Jun 2016, and all eligible studies from Oct 2015-June 2016, we classified growth metrics based on author-assigned labels (e.g., ‘weight gain’) and a ‘content signature’, a numeric code that summarized the metric’s conceptual and statistical properties. Heterogeneity was assessed by the number of unique content signatures, and label-to-content concordance. In 122 studies, we found 40 unique metrics of childhood growth. The most common approach to quantifying growth in length, weight or BMI was the calculation of each child’s change in z-score. Label-to-content discordance was common due to distinct content signatures carrying the same label, and because of instances in which the same content signature was assigned multiple different labels. In conclusion, the numerous distinct growth metrics and the lack of specificity in the application of metric labels challenge the integration of data and inferences from studies investigating the determinants or consequences of variations in childhood growth.

## Introduction

There is substantial ongoing investment in research into the early life factors that influence the development of chronic diseases such as obesity and cardiovascular disease. Of particular interest is the hypothesis that a child’s size at birth and the subsequent infant and early childhood growth (i.e., change in size over time) influence the risk of later metabolic and cardiovascular conditions [1]. Epidemiologic studies of the developmental origins of health and disease (DOHaD) hypothesis often rely on quantitative measures of early childhood growth that distinguish children with respect to their relative rates of growth (e.g., weight gain, length/height increases) during critical and sensitive windows of development. Studies are typically focused on growth as an exposure causing later childhood or adult conditions [2–12], or as an outcome caused by earlier factors [4,13–17].

The evidence that relatively slow versus fast growth in early life influences the risk of later health conditions has been conflicting [18]. Between-study inconsistencies may be largely due to differences in statistical methods, as exemplified by considering the variability among studies of the hypothesized association between early child growth and future blood pressure. First, a wide range of statistical models have been used to address the association between growth and other health outcomes; for example, a previous review of growth models demonstrated that different approaches (e.g., lifecourse plots and models versus latent growth curve models) can yield varying inferences regarding the association of growth with later systolic blood pressure [19]. Second, discrepancies in effect estimates can sometimes be attributed to subtle differences in the parameterization of repeated measures of size in regression models [20]; for example, regression models that adjust for current size [21,22] and those that condition on earlier measurements of size [23,24] yield different contrasts, but it can be challenging to reconcile the nuanced distinctions in the interpretations of the regression coefficients.

The varying definitions and statistical formulations used to quantify growth metrics may complicate efforts to integrate evidence across studies, particularly in the context of meta-analyses [4]. Recent reviews have narratively described the lack of a standardized approach for analyzing growth [25–28], yet no study to our knowledge has empirically characterized the extent of the definitional variation of growth in the recent published literature. Therefore, the specific objectives of this review were: 1) to generate an empirical framework for categorizing operational definitions of child growth, and 2) to use this framework to describe the range and frequency of metrics used to quantify early postnatal growth in recent epidemiological research.

## Methods

### Study inclusion and exclusion criteria

We conducted a scoping review to systematically summarize the variability in metrics of early childhood growth in recently published human growth research, following PRISMA (Preferred Reporting Items for Systematic Reviews and Meta-analyses) guidelines (S1 File) [29]. We sought to include peer-reviewed longitudinal studies published from January 2010 to June 2016 in which child growth was used as an exposure (independent) variable or outcome (dependent) variable and the analytical approach used ≥ 2 serial measures of length/height, weight or body mass index (BMI), with at least one measure taken in the period between birth to 5 years (up to and including 60 months of age). Multiple studies involving the same cohort were eligible for inclusion, as the metric of growth or age interval (i.e., timing of follow-up measures) can vary across published articles. We excluded: studies of animal growth, review articles or meta-analyses that did not present original individual-level analyses, studies involving only data simulations/mathematical models rather than empiric analyses of individual-level data, and studies that were published in any language other than English, as language is essential to data extraction and classification (attaching growth labels to definitions would be too complicated across multiple languages).

### Search strategy

MEDLINE and Embase electronic databases were searched for relevant articles in June 2016. The search syntax included a comprehensive list of keywords, medical subject heading (MeSH) (MEDLINE) and Emtree terms (EMBASE) identifying the study design, participant age group, anthropometric measure, and growth metrics (S2 File).

### Study selection

Study selection was conducted in two stages. The first stage consisted of title and abstract screening based on the eligibility criteria. Abstracts were excluded if they did not meet all the inclusion criteria. If there was insufficient data or if it was unclear from the title and abstract whether a study met the inclusion criteria, it was included in the full-text screening. In the second stage, we conducted full-text screening for 1) a 10% random sample of studies identified from the title and abstract screening, as it was unfeasible to full-text screen all articles given the large numbers identified in the electronic databases, and 2) all studies identified in the most recent 9 months of the search (October 2015 to June 2016) to ensure we did not miss any up-to-date strategies due to our random sampling approach.

For title/abstract and full-text screening, two reviewers independently screened each article using the web-based platform, COVIDENCE [30]. Any disagreements about inclusion/exclusion at the screening stage were flagged for a third reviewer to make the final decision on the eligibility of the study.

### Data abstraction

**Fig 1:**
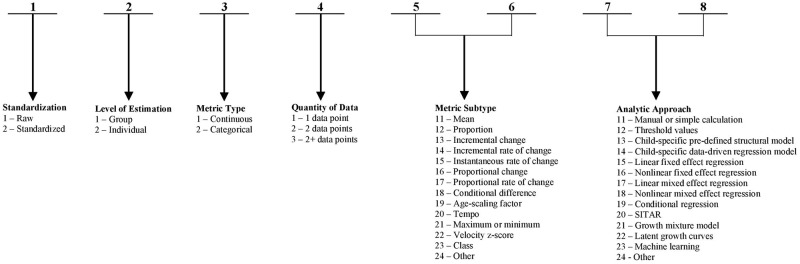
Components and ranges of possible values of the 8-digit content signature. Each component of the signature is represented by a 1- or 2-digit code, and the component codes were concatenated to generate the 8-digit content signature for each metric.

Data from eligible studies were abstracted using a standardized data abstraction tool designed for this study. The tool captured the relevant information on key study characteristics and detailed information on all metrics used to estimate/describe growth based on at least two data points per child/group (even though our tool can accommodate metrics based on cross-sectional analyses) anywhere in the article, including metrics that were mentioned in the narrative yet for which results were not shown. The metric ‘label’ was considered to be the word or phrase used by the study authors to identify a particular growth metric (e.g., “weight gain”, “length velocity”), where one metric could potentially have multiple labels. The metric ‘content’ consisted of the conceptual and statistical properties of the metric (i.e., the derivation/estimation, application and interpretation of the growth parameter), which we deconstructed into a 6-component (8 digit) ‘content signature’ (Fig 1): standardization (metric based on raw measurements or z-scores), level of analysis (individual or group as the unit of analysis), metric type (expressed as a continuous or categorical variable), quantity of data (minimum number of size measurements per individual/group that were used in the derivation of the growth metric), metric subtype (further classification of the manner by which the metric was quantified and expressed) and analytical approach (categorization, calculation or estimation method). As an example, Escribano et al. (2016) operationalized growth in weight as an incremental rate of change (grams per month) by taking the difference in unstandardized weight between birth and 6 months and dividing it by the duration of time, which they described as ‘weight gain velocity’ [31]. Using our framework (Fig 1), this metric would be classified as follows: ‘1-Raw’ for standardization, ‘2-Individual’ for level of estimation, ‘1-Continuous’ for metric type, ‘2–2 data points’ for quantity of data, ‘14-Incremental rate of change’ for metric subtype and ‘11-Manual or simple calculation’ for analytic approach. These component codes can then be concatenated to generate the content signature for this metric: ‘12121411’. More detailed descriptions of the 6-component framework can be found in S3 File.

Two reviewers independently extracted data from each eligible article. Any disagreements were resolved through discussion between the two reviewers or further adjudication by a third reviewer. Data abstraction was implemented using REDCap [32], a customizable informatics systems-based web software.

### Data analysis

We quantified the heterogeneity among growth metrics by comparing the relative frequency of use of each unique content signature in six strata defined by anthropometric parameter (length/height, weight or BMI) and whether the metric was used as an exposure or outcome variable in the growth analysis. In the stratified analyses, we also examined label-to-content concordance by constructing a matrix of the content signatures by the author-assigned labels. We also used the content signature components to construct decision trees to illustrate the most common approaches to growth analyses in the literature given the relative frequency of use of each unique metric based on content signatures only for a particular anthropometric parameter.

## Results

**Fig 2:**
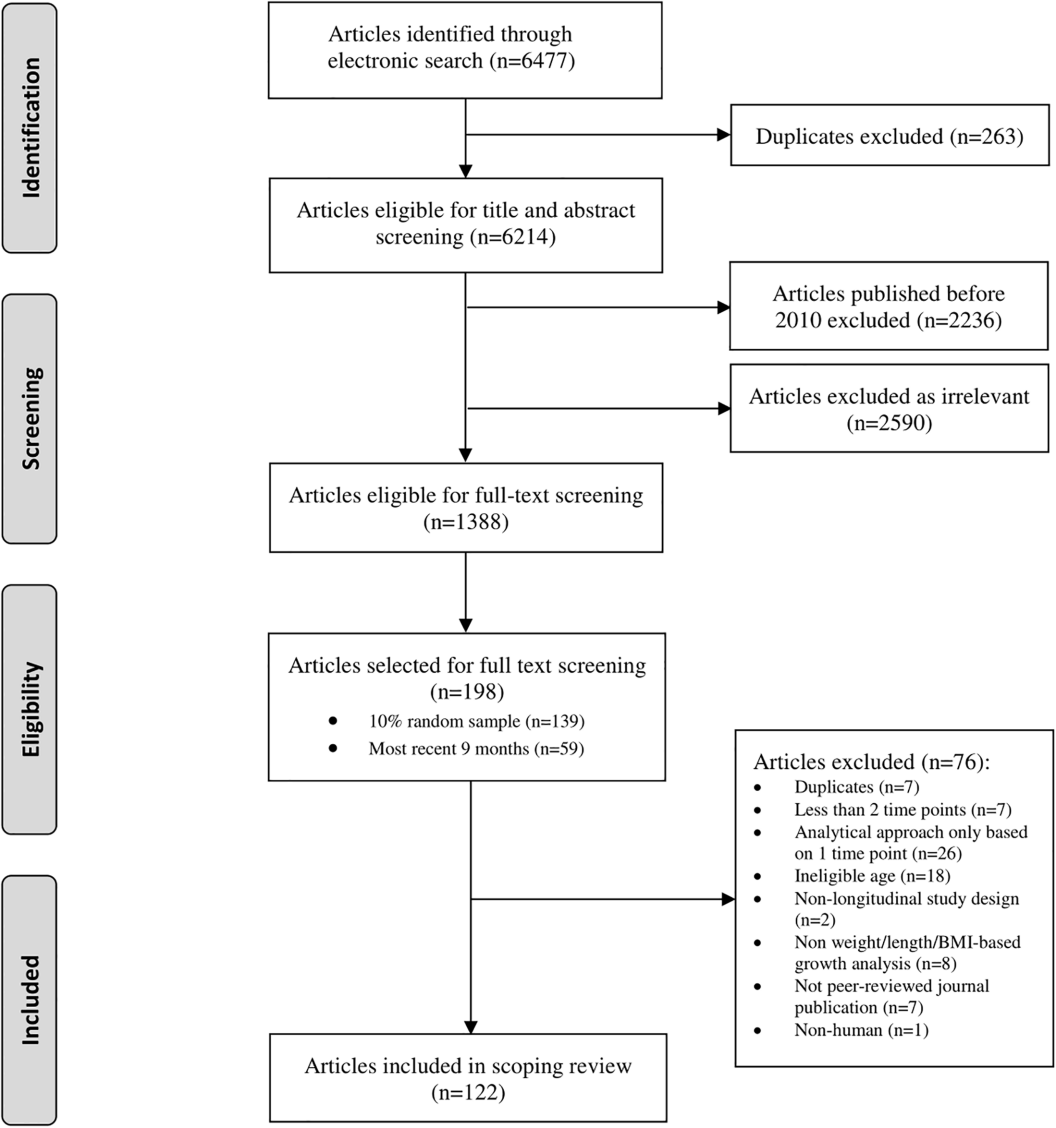
Flow of study selection.

Among 6477 articles retrieved from electronic databases, 122 studies of child growth were eligible and randomly selected for inclusion in the scoping review (Fig 2; S4 File for details). Most of the studies included in this review were cohort studies, conducted in the Americas or the European regions, and enrolled pregnant women or infants within the first month of life (Table 1).

**Table 1:** Characteristics of studies included in the scoping review of metrics of early childhood growth in epidemiological research.

Study Characteristics	All, *n* (%)	Length, *n* (%)	Weight, *n* (%)	BMI, *n* (%)
Total	122 (100)	64 (100)	78 (100)	39 (100)
Study design				
Cohort	82 (67)	40 (63)	48 (62)	26 (67)
Trial	36 (30)	23 (36)	27 (35)	11 (28)
Other^a^	4 (3)	1 (2)	3 (4)	2 (5)
Age of earliest enrolment				
Prenatal	20 (16)	12 (19)	14 (18)	4 (10)
0–1 month	44 (36)	17 (27)	32 (41)	10 (26)
1–24 months	30 (25)	20 (31)	23 (30)	10 (26)
24–60 months	28 (23)	15 (23)	9 (12)	15 (38)
Sample size				
<100	34 (28)	15 (23)	21 (27)	10 (26)
100–500	39 (32)	21 (33)	27 (35)	10 (26)
>500	49 (40)	28 (44)	30 (38)	19 (49)
Region of study population^b^				
African	8 (7)	7 (11)	8 (10)	0 (0)
Americas	38 (31)	17 (27)	19 (24)	17 (44)
South East Asia	6 (5)	4 (6)	4 (5)	1 (3)
European	48 (39)	24 (38)	32 (41)	15 (39)
Eastern Mediterranean	2 (2)	1 (2)	1 (1)	0 (0)
Western Pacific	16 (13)	8 (13)	12 (15)	6 (15)
Multiple	4 (3)	3 (5)	2 (3)	0 (0)
Publication year				
2010	11 (9)	6 (9)	6 (8)	3 (8)
2011	10 (8)	4 (6)	6 (8)	3 (8)
2012	26 (21)	17 (27)	14 (18)	9 (23)
2013	10 (8)	7 (11)	7 (9)	2 (5)
2014	26 (21)	11 (17)	18 (23)	7 (18)
2015	29 (24)	15 (23)	18 (23)	13 (33)
2016^c^	10 (8)	4 (6)	9 (12)	2 (5)
Number of growth metrics reported per study				
1 growth metric	60 (49)	45 (70)	60 (77)	33 (85)
2 growth metrics	36 (30)	16 (25)	15 (19)	3 (8)
3+ growth metrics	26 (21)	3 (5)	3 (4)	3 (8)

^a^ Other study designs include retrospective chart reviews (n = 3) and non-randomized interventional cohorts (n = 1)

^b^ Based on WHO classifications

^c^ The search strategy was last performed on June 2, 2016 and therefore did not include all of 2016.

**Fig 3:**
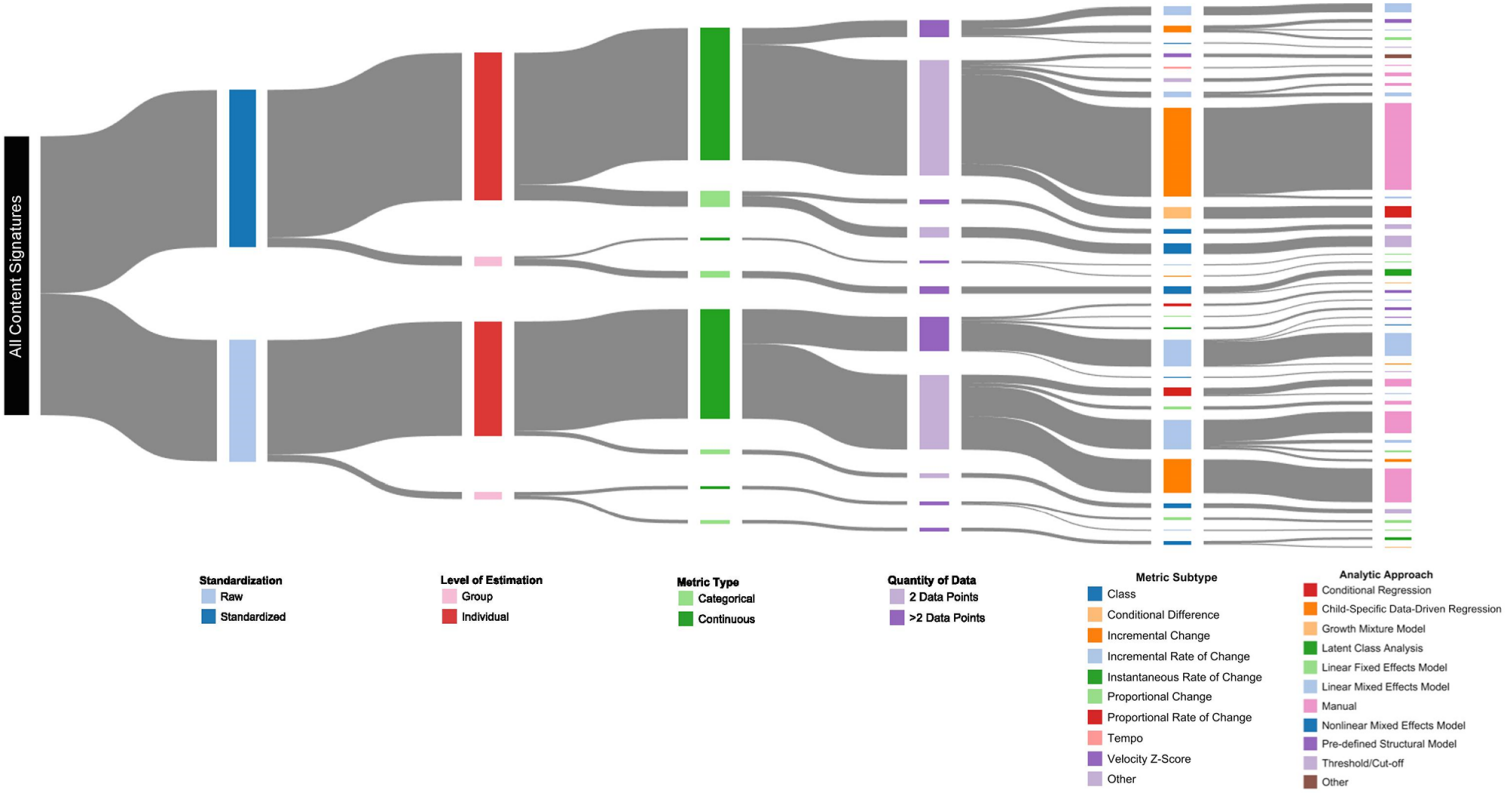
A Sankey diagram to illustrate the heterogeneity among published metrics for child growth in length, weight or body mass index (n = 235) and relative prevalences overall and within each component. Moving from left to right, content signatures are deconstructed into their individual components (i.e., standardization, level of estimation, metric type, quantity of data, metric subtype, analytic approach), where the width of the band is proportional to the frequency of the approach. The most common approach was the calculation of each child’s incremental change in the standardized anthropometric parameter, which is represented by the band that flows through the following nodes: ‘standardized parameter’ (dark blue), ‘individual level of analysis’ (dark red), ‘continuous variable’ (dark green), ‘2 data points’ (light purple), ‘incremental change’ (dark orange), and ‘manual calculation’ (pink). The range of growth metrics presented is based on a random sample of published studies, and therefore is not exhaustive.

**Fig 4:**
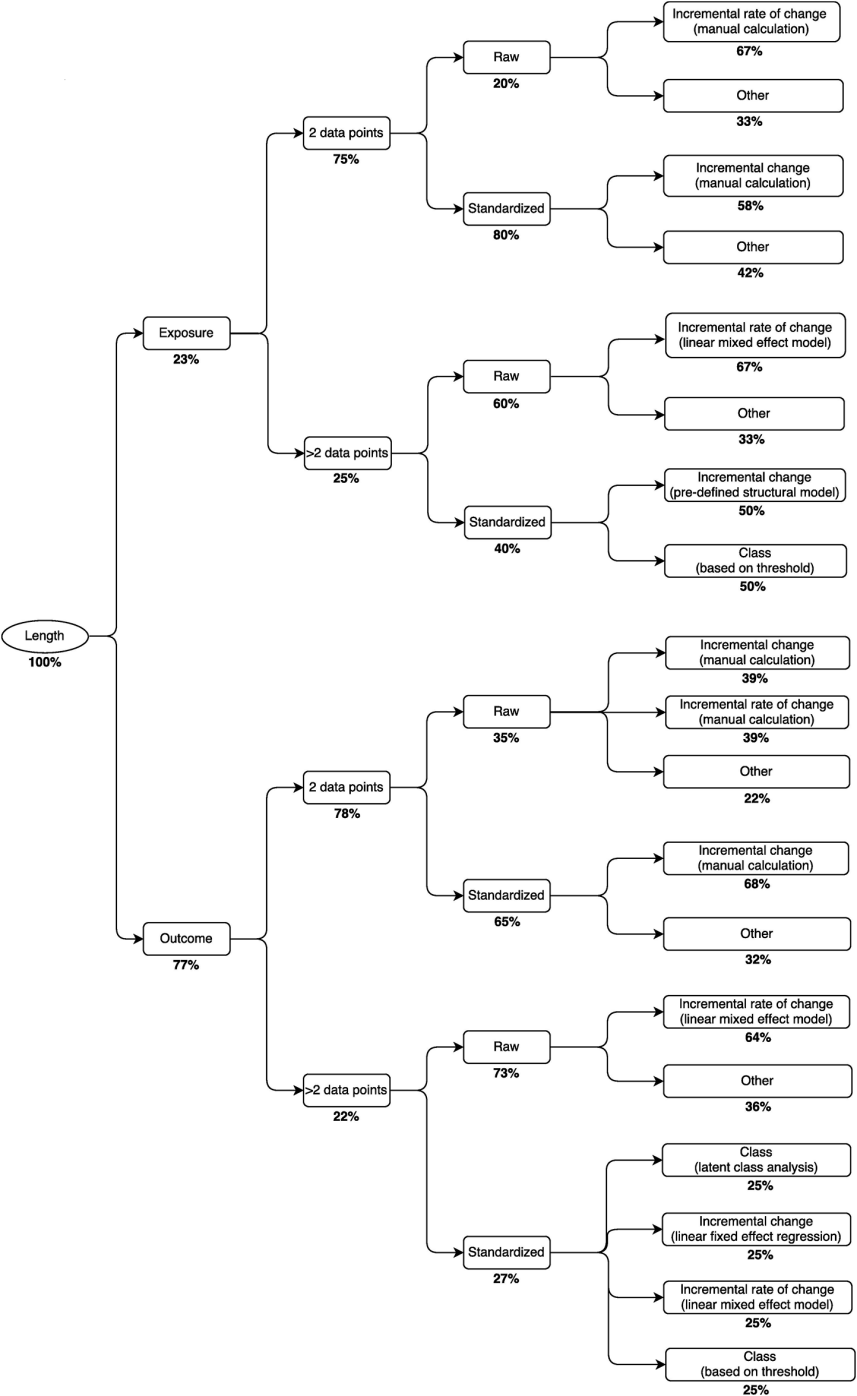
Decision tree for selection of metrics of growth in length (n = 87). Percentages represent the relative prevalence of the approach at each branching point. For example, the most common approach for growth in length as an exposure with 2 data points is to first standardize the anthropometric parameter, then calculate the incremental change.

**Fig 5:**
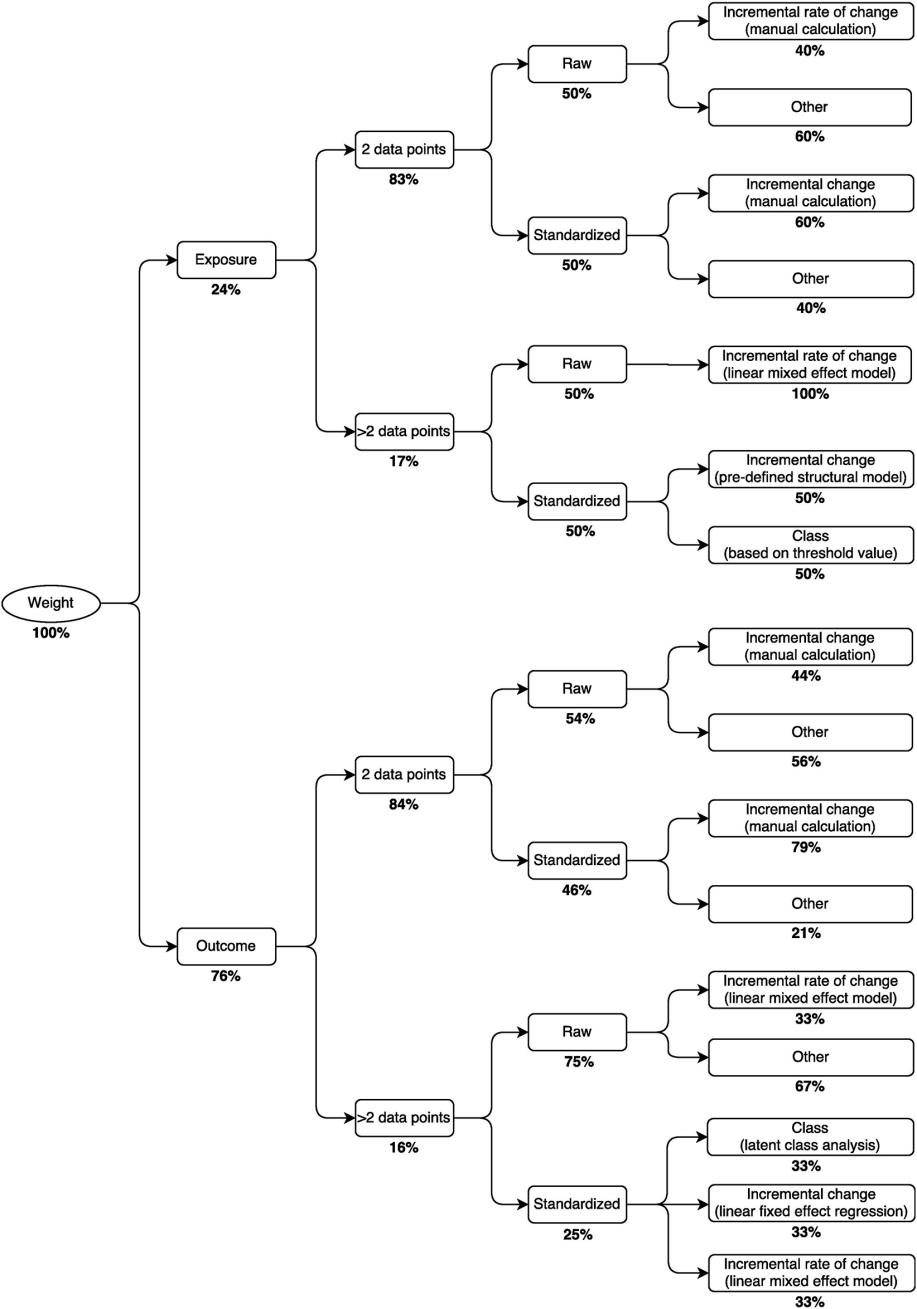
Decision tree for selection of metrics of growth in weight (n = 99). Percentages represent the relative prevalence of the approach at each branching point. For example, the most common approach for estimating growth in weight as an outcome with >2 data points was to calculate the incremental rate of change of unstandardized weight using a linear mixed effects model.

**Fig 6:**
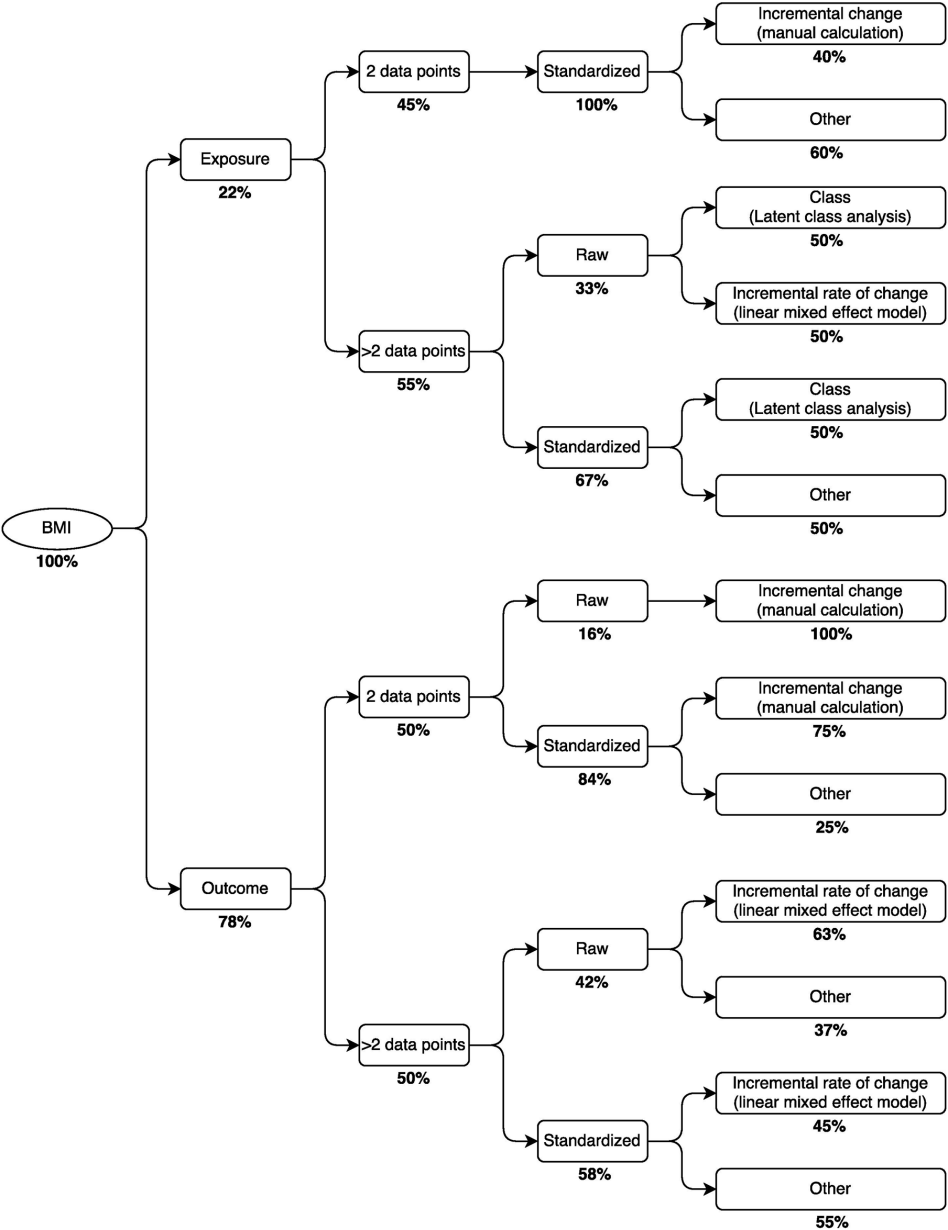
Decision tree for selection of metrics of growth in BMI (n = 49). Percentages represent the relative prevalence of the approach at each branching point. For example, the most common approach for expressing growth in BMI as an exposure with >2 data points was to first standardize BMI, then analyze it in relation to an outcome using latent class analysis.

In the 122 included articles, we identified a total of 235 early childhood growth metrics, among which there were 40 unique metrics based on content signatures (Fig 3). There was substantial overlap in the use of these 40 metrics across the three anthropometric parameters (length, weight, BMI) and between exposure and outcome variables. Of the 40 unique metrics, 3 were only used as exposure variables, 24 were only used as outcome variables, and 13 were used at least once as both exposure and outcome variables. Among 16 unique metrics used at least once as an exposure variable, 2 were used only for length, 3 only for weight, 3 only for BMI, and 8 for more than one anthropometric parameter. Among 37 unique metrics used at least once as an outcome variable, 3 were used only for length, 7 only for weight, 8 only for BMI, and 19 for more than one anthropometric parameter. All unique content signatures are listed in S5 File, and decision trees constructed on the basis of the content signature components are shown in Figs 4–6.

Overall, the most common approach to quantifying growth (31% of all 235 metrics) was a simple calculation of each child’s incremental change (i.e., arithmetic difference in size between two time points spanning a specified interval) using a standardized expression of the anthropometric parameter (i.e., z-score) (Fig 3). This finding remained consistent when stratifying by the anthropometric parameter (i.e., length, weight or BMI) and whether the metric was used as an exposure or outcome (Tables 2 and 3). The second most common approaches varied across the different strata defined by parameter and usage as an exposure/outcome variable. For example, the second most frequently used metrics for growth in length and weight (as an exposure) were conditional growth in standardized length (15%) and the incremental rate of change in unstandardized weight (17%), respectively (Table 2). For other strata, there were no other dominant approaches; e.g., for length and BMI as an outcome, three different content signatures were used 11% of the time, and two different content signatures were used 13% of the time, respectively (Table 3).

**Table 2:** Common content signatures and their associated author-specified labels for growth as an exposure, by anthropometric parameter^a^.

Parameter	n/N^b^ (%)	Signature description	Author-specified labels
Signature			
Length			
22121311	7/20 (35)	Estimation of the incremental change in standardized anthropometric parameters between 2 time points using simple/manual calculation	change, gain, growth, linear growth
22121819	3/20 (15)	Estimation of the conditional change in standardized anthropometric parameters between 2 time points using a conditional regression (residual estimated by regressing current height-for-age-z-score (HAZ) on previous HAZ)	conditional change, conditional gain, conditional growth, gain, growth, growth trajectory, linear growth, velocity
12121411	2/20 (10)	Estimation of the incremental rate of change in unstandardized anthropometric parameters between 2 times points using simple/manual calculation	gain velocity, velocity
12131417	2/20 (10)	Estimation of the incremental rate of change in unstandardized anthropometric parameters on the basis of >2 data points using a linear mixed effects model	growth, growth trajectory, linear growth, rate of growth
Weight			
22121311	6/24 (25)	Estimation of the incremental change in standardized anthropometric parameters between 2 time points using simple/manual calculation	change, gain, growth, growth velocity
12121411	4/24 (17)	Estimation of the incremental rate of change in unstandardized anthropometric parameters between 2 times points using simple/manual calculation	gain, gain rate, gain velocity
12222312	3/24 (13)	Creation of classes in unstandardized anthropometric parameters using threshold values with 2 data points	gain, growth
BMI			
22121311	2/11 (19)	Estimation of the incremental change in standardized anthropometric parameters between 2 time points using simple/manual calculation	change, gain
21232322	2/11 (18)	Creation of classes in standardized anthropometric parameters on the basis of >2 data points using latent class analysis	growth, growth trajectory class, longitudinal growth, pattern of change, trajectory class, trajectory group, trajectory pattern class, velocity

^a^ ‘Common’ refers to the 3 most frequently used signatures, excluding any signatures that were used only once

^b^ ‘n’ refers to the number of times the metric was used, ‘N’ refers to the total number of metrics, and the % reflect the prevalence of the content signature

**Table 3:** Common content signatures and their associated author-specified labels for growth as an outcome, by anthropometric parameter^a^.

Parameter	n/N^b^ (%)	Signature description	Author-specified labels
Signature			
Length			
22121311	23/67 (34)	Estimation of the incremental change in standardized anthropometric parameters between 2 time points using simple/manual calculation	catch-up growth, change, deficit, difference, gain, growth, improvement, rate, velocity
12121311	7/67 (11)	Estimation of the incremental change in unstandardized anthropometric parameters between 2 time points using simple/manual calculation	change, difference, gain, growth, increment
12121411	7/67 (11)	Estimation of the incremental rate of change in unstandardized anthropometric parameters between 2 times points using simple/manual calculation	gain, growth, growth rate, growth velocity, linear growth velocity, trajectory, velocity
12131417	7/67 (11)	Estimation of the incremental rate of change in unstandardized anthropometric parameters on the basis of >2 data points using a linear mixed effects model	change, growth, growth rate, growth trajectory, growth velocity, linear growth, rate of growth
22121819	3/67 (5)	Estimation of the conditional change in standardized anthropometric parameters between 2 time points using a conditional regression (residual estimated by regressing current height-for-age-z-score (HAZ) on previous HAZ)	conditional change, conditional growth velocity, conditional velocity, growth, growth trajectory, linear growth, velocity
22222312	3/67 (5)	Creation of classes in standardized anthropometric parameters using threshold values with 2 data points	catch-down growth, catch-up growth, change, growth, growth pattern, recovery from stunting
Weight			
22121311	23/75 (31)	Estimation of the incremental change in standardized anthropometric parameters between 2 time points using simple/manual calculation	catch-up growth, change, difference, gain, growth, growth pattern, growth rate, improvement
12121311	15/75 (20)	Estimation of the incremental change in unstandardized anthropometric parameters between 2 time points using simple/manual calculation	change, delta, difference, gain, growth, increment
12121711	6/75 (8)	Estimation of the proportional rate of change in unstandardized anthropometric parameters between 2 time points using simple/manual calculation	fractional growth rate, gain, gain velocity, growth, growth velocity
BMI			
22121311	12/38 (32)	Estimation of the incremental change in standardized anthropometric parameters between 2 time points using simple/manual calculation	change, delta, difference, gain, growth
12131417	5/38 (13)	Estimation of the incremental rate of change in unstandardized anthropometric parameters on the basis of >2 data points using a linear mixed effects model	change, growth trajectory, rate of change, rate of growth, trajectory
22131417	5/38 (13)	Estimation of the incremental rate of change in standardized anthropometric parameters on the basis of >2 data points using a linear mixed effects model	change, change over time, gain, growth, rate of change, rate of gain, rate of weight gain, trajectory, trend, velocity
12121311	3/38 (8)	Estimation of the incremental change in unstandardized anthropometric parameters between 2 time points using simple/manual calculation	change, change score, difference, gain, growth pattern

^a^ ‘Common’ refers to the 3 most frequently used signatures, excluding any signatures that were used only once

^b^ ‘n’ refers to the number of times the metric was used, ‘N’ refers to the total number of metrics, and the % reflect the prevalence of the content signature

Overall, few studies specifically derived ‘conditional’ growth metrics (10/235; 4% of all metrics), referring to model residuals from the regression of size at the end of an interval on size at the beginning of the interval; the majority of these metrics were applied to length (6 of the 10 uses of conditional metrics). However, in studies that generated ‘unconditional’ metrics (e.g., change in z-score between two time points), 31% of these metrics were subsequently used in regression models in which there was adjustment for size at the beginning of the age interval of interest, thereby ultimately generating estimates that were conditional on baseline/earlier size. Notably, only 6 of the total 122 studies in the review (5%) explicitly reported any methodological considerations for regression to the mean when examining growth using longitudinal data.

Label-to-content discordance was common due to distinct signatures carrying the same author-assigned label, and because of differently assigned labels to the same content signature between authors (Tables 2 and 3). For example, the most common 8-digit signature for growth in length as an exposure (22121311; incremental change between 2 points) was labeled as ‘change’, ‘gain’, and ‘growth’ (Table 2). However, these same labels were also commonly used to refer to the second most common signature for growth in BMI as an outcome (22131417; child-specific rate of change estimated from a linear mixed model) (Table 3). Label-to-content matrices can be found in S6 File.

## Discussion

In the present scoping review, we found that a diverse array of statistical metrics has been used in recent published literature to quantify early childhood growth. Metrics with simple derivations, such as the estimation of the incremental change in an anthropometric parameter between two time points, were much more commonly used than those that require either more complex statistical methods, such as latent class analysis, or a deeper understanding of the theoretical assumptions required to make inferences, such as conditional growth models.

Investigators in the field of human growth research are often aware of the nuances that influence the selection of particular analytical approaches that best suit the research question. However, we found that an explicit justification of the choice of approach—e.g., raw BMI “is more appropriate for analyzing change over time” [33], or the use of the Berkey-Reed 1^st^-order model to “reflect the actual pattern of change that child health practitioners will observe” [34]–was the exception rather than the norm. That is, specific growth model selection was not well justified with a narrative rationale, even if investigators selected a suitable analytical approach to address their research question (e.g., the use of conditional growth models to investigate independent associations between consecutive growth periods and later health outcomes).

In principle, the use of distinct metrics or statistical approaches to growth analyses may partly explain the inconsistent findings relating child growth to later health and economic outcomes. For example, the adjustment for size at the beginning of the growth interval in a regression model or the use of the classical formulation of ‘conditional growth’ [23,35–37] (two approaches that are algebraically interchangeable) yield estimates of growth effects that are conditional on baseline size; therefore, inferences from these models are expected to differ from analyses of the same growth-outcome association in which there was no adjustment for baseline or earlier size [38]. Conversely, many growth metrics that appear superficially distinct (and were assigned different content signatures in our analysis) are in fact interchangeable re-parameterizations of the longitudinal data, such that the between-child variance in growth may be similarly captured by the different approaches. For example, inferences from an incremental change over time calculated manually may be similar to a child-specific slope derived using a linear mixed effects model.

We found wide variability in label and content signature combinations. Many content signatures were associated with the same label, and there were also instances in which the same content signature was assigned multiple different labels (S6 File). Many commonly used generic labels (e.g., growth, gain) are suitably applied to a range of metrics, but lack precision. The use of the term ‘velocity’ was widely used with highly variable meanings. For example, a conventional use of the term refers to a ‘rate of change’ implying a denominator that represents a time interval; however, in other cases it was used to describe changes in z-scores, yet since z-scores are centered on zero rather than being consistently positive, the term ‘velocity’ may be less intuitive when used to quantify the extent to which a child’s growth curve deviates from the trajectory predicted on the basis of a population growth reference/standard. The discordance between metric labels and their statistical formulations poses a particular methodological challenge for systematic reviews and meta-analyses, in which the terms used in a search strategy may not fully capture the true scope of the relevant literature. For instance, a systematic review summarizing the effect of probiotics on child growth only used the terms ‘growth’ and ‘stunt’ in their search strategy [39], while another review assessing factors associated with accelerated growth in childhood only used the terms ‘catch-up’ and ‘rapid weight gain’ [40]. The limited range of search terms used by investigators may bias the selection of studies for inclusion, and therefore may have implications for evidence synthesis.

The content signatures that we designed to classify growth metrics in this review may be used to formulate decision trees to inform investigators of the most common approaches to growth analyses in the literature, given the particular anthropometric parameter of interest and the data available for analysis (Figs 4–6). For example, in a study to investigate early growth in length as a risk factor for a future health outcome, such as blood pressure in mid-childhood, whereby length was assessed at only two time points across the age interval of interest, it may be instructive for investigators to know that most previous studies with the same data structure standardized the anthropometric parameter and then calculated an incremental change (Fig 4). Alternatively, in a study to examine growth in weight in relation to a set of antecedent risk factors, in which weight is assessed at more than two time points in the age interval of interest, the most commonly used approach among previous studies was to estimate the incremental rate of change in the unstandardized anthropometric parameter using a linear mixed effect model (Fig 5). The routine reporting of analyses using the most common metric, if it appropriately addresses the question of interest, may promote more straightforward comparisons and synthesis of results across studies, even if authors additionally report other less-common or novel analytical approaches that they consider to be particularly suited to their research question or study design. However, we also suggest that authors be as explicit as possible with regards to their research question and provide suitable justification for their specific choice of growth metric and modeling approach, so that the coherent fit of the modeling approach to the research question is apparent.

Several limitations of the review should be acknowledged. First, we may have missed recent or relatively uncommon methods used to analyze early childhood growth due to our sampling approach as it was not feasible to screen and data abstract from all published articles given the large numbers identified in the electronic databases and the laborious process of extracting and classifying each metric. For example, the SITAR method [41–43] or the use of WHO velocity charts [44] have been used in some recent studies, but due to the low frequency of their uses, they did not appear in our random selection of studies. However, since our pool of studies is comprised of a random sample, we considered it to be a fair representation of variability with which researchers currently operationalize and quantify growth. Another weakness of the review was our focus on growth in length, weight and BMI only, yet there are numerous other anthropometric parameters that may be relevant to human growth research (e.g., head circumference, weight-for-length, ponderal index, skin-fold thickness), for which there may be different content signatures. Thus, we may have underestimated the true heterogeneity in growth metrics in the recent literature. Finally, our classification of growth metrics was based on six components; there may be other relevant components that we did not incorporate in our analysis that would further differentiate metrics.

In summary, this scoping review was not designed to identify a set of ideal metrics to summarize growth, as the choice of growth model is contingent on both available data and the specific research question. However, our findings indicate the need for greater consensus on standardized approaches to summarizing growth for specific questions of interest. Variations in growth metrics complicate comparisons of findings across studies, and discordance between metric labels and their statistical formulation further challenges the integration of inferences. We conclude that the implications of child growth metric heterogeneity should be considered when aggregating and/or designing studies of the causal determinants or consequences of variations in early childhood growth.

## Supporting information

S1 File Supporting information A. PRISMA Checklist.
(DOC)

S2 File Supporting information B. Electronic Database Search Strategy.
(DOCX)

S3 File Supporting information C. Growth Metric Content Signature Component Definitions.
(DOCX)

S4 File Supporting information D. Studies included in the review.
(DOCX)

S5 File Supporting information E. Content Signatures.
(DOCX)

S6 File Supporting information F. Label-to-content matrices.
(DOCX)
