# Supplementary material for: Metrics of early childhood growth in recent epidemiological research: A scoping review
Source: PLoS One. 2018 Mar 20;13(3):e0194565. doi: 10.1371/journal.pone.0194565 (PMC5860780; doi:10.1371/journal.pone.0194565)
Supplement: S2 File — Electronic Database Search Strategy. (DOCX) [file pone.0194565.s002.docx]

# **B. Electronic Database Search Strategy**

**MEDLINE**^a^

| 1 length.tw,kf.  2 height.tw,kf.  3 weight.tw,kf.  4 size.tw,kf.  5 stature.tw,kf.  6 bmi.tw,kf.  7 "body mass index".tw,kf.  8 "head circumference".tw,kf.  9 "body composition".tw,kf.  10 anthropometr*.tw,kf.  11 stunt*.tw,kf.  12 underweight.tw,kf.  13 overweight.tw,kf.  14 1 or 2 or 3 or 4 or 5 or 6 or 7 or 8 or 9 or 10 or 11 or 12 or 13  15 grow*.tw,kf.  16 chang*.tw,kf.  17 velocit*.tw,kf.  18 trajector*.tw,kf.  19 pattern*.tw,kf.  20 gain*.tw,kf.  21 rate*.tw,kf.  22 slope*.tw,kf.  23 falter*.tw,kf.  24 increment*.tw,kf.  25 accretion*.tw,kf.  26 accrual*.tw,kf.  27 curve*.tw,kf.  28 ("catch-up" or "catch up").tw,kf.  29 ("catch-down" or "catch down").tw,kf.  30 15 or 16 or 17 or 18 or 19 or 20 or 21 or 22 or 23 or 24 or 25 or 26 or 27 or 28 or 29  31 ((length or height or weight or size or stature or bmi or "body mass index" or "head circumference" or "body composition" or anthropometr* or stunt* or underweight or overweight) adj3 (grow* or chang* or velocit* or trajector* or pattern* or gain* or rate* or slope* or falter* or increment* or accretion* or accrual* or curve* or ("catch-up" or "catch up") or ("catch-down" or "catch down"))).tw,kf.  32 (grow* adj3 falter*).tw,kf.  33 (grow* adj3 (trajector* or velocit* or pattern* or curve*)).tw,kf.  34 31 or 32 or 33  35 infan*.tw,kf.  36 child*.tw,kf.  37 toddler*.tw,kf.  38 preschool*.tw,kf.  39 (baby or babies).tw,kf.  40 (boy* or girl*).tw,kf.  41 postnatal.tw,kf.  42 35 or 36 or 37 or 38 or 39 or 40 or 41  43 34 and 42  44 cohort studies/ or longitudinal studies/ or follow-up studies/ or observational studies/ or prospective studies/ or retrospective studies/  45 randomized controlled trial.pt.  46 controlled clinical trial.pt.  47 placebo.ab.  48 clinical trials as topic.sh.  49 randomly.ab.  50 trial.ti.  51 45 or 46 or 47 or 48 or 49 or 50  52 51 not 44  53 review.pt.  54 "systematic review".pt.  55 53 or 54  56 52 not 55  57 43 and 56  58 exp animals/ not humans.sh.  59 57 not 58  60 cross-sectional studies/ or case-control studies/  61 59 not 60  62 limit 61 to english language |
| --- |

^a^ Head circumference was included to increase the sensitivity of the search for any articles related to child growth/anthropometry, even though it was not analyzed as an anthropometric parameter in the review.

**EMBASE**^a^

| 1 length.tw.  2 height.tw.  3 weight.tw.  4 size.tw.  5 stature.tw.  6 bmi.tw.  7 "body mass index".tw.  8 "head circumference".tw.  9 "body composition".tw.  10 anthropometr*.tw.  11 stunt*.tw.  12 underweight.tw.  13 overweight.tw.  14 1 or 2 or 3 or 4 or 5 or 6 or 7 or 8 or 9 or 10 or 11 or 12 or 13  15 grow*.tw.  16 chang*.tw.  17 velocit*.tw.  18 trajector*.tw.  19 pattern*.tw.  20 gain*.tw.  21 curve*.tw.  22 rate*.tw.  23 slope*.tw.  24 falter*.tw.  25 increment*.tw.  26 accretion*.tw.  27 accrual*.tw.  28 ("catch-up" or "catch up").tw.  29 ("catch-down" or "catch down").tw.  30 15 or 16 or 17 or 18 or 19 or 20 or 21 or 22 or 23 or 24 or 25 or 26 or 27 or 28 or 29  31 ((length or height or weight or size or stature or bmi or "body mass index" or "head circumference" or "body composition" or anthropometr* or stunt* or underweight or overweight) adj3 (grow* or chang* or velocit* or trajector* or pattern* or gain* or curve* or rate* or slope* or falter* or increment* or accretion* or accrual* or ("catch-up" or "catch up") or ("catch-down" or "catch down"))).tw.  32 (grow* adj3 falter*).tw.  33 (grow* adj3 (trajector* or velocit* or pattern* or rate* or curve*)).tw.  34 31 or 32 or 33  35 infan*.tw.  36 child*.tw.  37 toddler*.tw.  38 preschool*.tw.  39 (baby or babies).tw.  40 (boy* or girl*).tw.  41 postnatal.tw.  42 35 or 36 or 37 or 38 or 39 or 40 or 41  43 34 and 42  44 ("cohort stud*" or "longitudinal stud*" or "follow-up stud*" or "observational stud*" or "prospective stud*" or "retrospective stud*").tw.  45 randomized controlled trial.pt.  46 controlled clinical trial.pt.  47 placebo.ab.  48 randomly.ab.  49 clinical trials as topic.sh.  50 trial.ti.  51 45 or 46 or 47 or 48 or 49 or 50  52 51 not 44  53 review.pt.  54 "systematic review".pt.  55 53 or 54  56 52 not 55  57 43 and 56  58 exp animals/ not humans.sh.  59 57 not 58  60 ("case-control studies" or "cross-sectional studies").tw.  61 59 not 60  62 limit 61 to english language |
| --- |

^a^ Head circumference was included to increase the sensitivity of the search for any articles related to child growth/anthropometry, even though it was not analyzed as an anthropometric parameter in the review.
