# Supplementary material for: Metrics of early childhood growth in recent epidemiological research: A scoping review
Source: PLoS One. 2018 Mar 20;13(3):e0194565. doi: 10.1371/journal.pone.0194565 (PMC5860780; doi:10.1371/journal.pone.0194565)
Supplement: S3 File — Growth Metric Content Signature Component Definitions. (DOCX) [file pone.0194565.s003.docx]

# **C. Growth Metric Content Signature Component Definitions**

## **Component I.** Standardization of anthropometric parameters

| **Selection** | **Description** | **Code** |
| --- | --- | --- |
| Raw/Unstandardized | - Anthropometric parameter was untransformed/unstandardized. Analysis was conducted on raw measures. For example, growth in length was analyzed in cm, weight in g and BMI in kg/m^2^. | 1 |
| Standardized | - Anthropometric parameter is expressed in standard deviation score or percentile relative to the population mean. For example, the reference population used for standardization may be: 1) the study population (i.e., internal standardization), 2) a representative country population (i.e., country-specific standards), 3) a multi-ethnic population (i.e., the WHO-GS or INTERGROWTH-21^st^ standards) | 2 |

## **Component II.** Level of analysis at which the metric was estimated

| **Selection** | **Description** | **Code** |
| --- | --- | --- |
| Group | - Group-level analyses, based on comparing the average growth trajectories among 2 or more groups defined by an exposure or outcome other than size/growth (e.g., treatment group in a trial). - Child-specific trajectories or velocities were not calculated, estimated or predicted in any analyses - Children were not categorized on the basis of their individual trajectory | 1 |
| Individual | - Child-specific trajectories were calculated, estimated or predicted for use in subsequent analyses. | 2 |

## **Component III.** Metric Type (Level at which metric was used)

| **Selection** | **Description** | **Code** |
| --- | --- | --- |
| Continuous | - Group- or individual-level descriptor of growth is a continuous variable that quantitatively ranks children in terms of faster or slower growth relative to peers - E.g., velocity, time to peak z-score, area-under-the-curve | 1 |
| Categorical | - Group- or individual-level description of growth is based on assignment to a class or category of growth trajectories. - This code should be selected even if the assignment to a category is based on an underlying continuous metric with a specified cut-off (e.g., ‘fast growth’ is based on a change in z-score of >0.67 z-scores). If the continuous metric is reported in both quantitative and categorical terms, these should be reported as distinct metrics. | 2 |

**Component IV.** Quantity of data upon which the metric was based*****

| **Selection** | **Description** | **Code** |
| --- | --- | --- |
| 1 data point | - Growth was described based on only one anthropometric data point per child (cross-sectional analysis) - For a group-average metric, this would be the mean of a group of children’s values - This code would apply even if the analysis involves serial cross-sectional analyses with only qualitative comments about changes over time. | 1 |
| 2 data points | - Each child or group trajectory or velocity was calculated, estimated, predicted or categorized based on a maximum of 2 size measurements, one at or near the beginning of the interval and one at or near the end of the interval | 2 |
| More than 2 points | - Each child or group trajectory or velocity was calculated, estimated, predicted or categorized based on 3 or more size measurements within an interval of interest. - This code should be used if the intention of the investigators was to use >2 data points, even if some of the children in the dataset did not (or could not be confirmed to have had) at least 3 data points in each interval. | 3 |

*For this component, if two different metrics were used to estimate growth over the same interval, but used varying number of data points, we only extracted details for the metric with more data points (e.g., if a study uses both LAZ at 1 year and incremental change in LAZ from 0-1 year in the analyses, we only included information on the latter).

## **Component V.** Metric Sub-Type*

| **Selection** | **Description** | **Code** |
| --- | --- | --- |
| Mean | - This would be the mean of a group of children’s values for a continuous variable | 11 |
| Proportion | - Prevalence or incidence of a group of children’s values for a categorical variable. | 12 |
| Incremental change | - Arithmetic difference between size at the end and beginning of a specified age/time interval - May be estimated at the individual or group level. - For group-level metrics, this can be a change in the group’s mean size, or the average of within-child changes. | 13 |
| Incremental rate of change | - Arithmetic difference between size at the end and beginning of a specified age/time interval, expressed as a function of time (velocity or rate, e.g. cm/year) - For metrics that were based on >2 data points per child, then incremental rate of change would be based on a slope representing the average rate of change of size over the specified interval. - The slope could represent an individual child OR a group mean. - Child-specific slopes may be expressed in absolute terms or relative to a group mean (e.g., a child-specific random slope, or best linear unbiased prediction, indicating a deviation from the group fixed effect). | 14 |
| Instantaneous rate of change | - Expressed similarly to the incremental rate of change, but based on the first-derivative at a single specified point on the size-by-age slope. - The slope from which the instantaneous rate of change is derived could represent an individual child OR a group mean trajectory. | 15 |
| Proportional change | - Fractional change (%) in size from the beginning to end of interval, relative to the child’s size at the beginning of the interval - Note: the exponentiated difference on the log-scale is the same as a proportional difference | 16 |
| Proportional rate of change | - Fractional change (%) in size from the beginning to end of interval, relative to the child’s size at the beginning of the interval, expressed as a function of time (e.g., % per month). - For metrics that were based on >2 data points per child, then proportional rate of change would be based on a child-specific linear slope representing the rate of change of *log-transformed* size over the specified interval. - The slope could represent an individual child OR a group mean. - Child-specific slopes may be expressed in absolute terms or relative to a group mean (e.g., a child-specific random slope as a deviation from the group fixed effect). | 17 |
| Conditional change (or conditional difference) | - Difference between the observed and expected size at the end of the interval, where the expected value is based on the absolute size at the beginning of the interval and the overall correlation between size at the beginning and end of the interval (within the group as a whole). - Usually this is an individual-level metric (therefore, be cautious about applying this descriptor to a group-level metric). - For studies in which growth is an exposure variable, conditional growth is typically estimated as the child-specific residual from a regression model in which size at a given age is regressed on size as an earlier age. - In studies in which growth is the outcome variable, size at the beginning of the interval may be included as one of several covariates in a regression model designed to identify predictors of (or risk factors for) growth. | 18 |
| Age-scaling factor | - Rate of growth is expressed in terms of a *proportional* expansion or contraction of the age scale | 19 |
| Tempo (time-to-event) | - Growth is described quantitatively in terms of the duration of a specific interval of interest, for which the end marks a definable event. - This metric may be derived directly from the size-by-age curve or from the velocity-by-age curve (e.g., time from birth to peak height velocity). | 20 |
| Maximum or minimum point on a trajectory | - A child or group trajectory is described in terms of the highest or lowest value on either the size-by-age or velocity-by-age curve. | 21 |
| Velocity z-score | - Use of an external reference or standard to assign a child-specific z-score to reflect *rate* of growth rather than size (e.g., the World Health Organization growth velocity z-scores) | 22 |
| Class | - Group- or individual-level description of growth is based on assignment to a class or category of growth trajectories. | 23 |
| Other | - Any other quantitative measure not mentioned above | 24 |

## **Component VI.** Analytical approach from which metric was derived

| **Selection** | **Description** | **Code** |
| --- | --- | --- |
| Manual or simple calculation | - Metric is based on the simple arithmetic operation for each child/group. - Did not involve statistical modeling. - Metric was derived empirically, rather than modeled or predicted. - This could apply to either quantitative or categorical metrics, if the above criteria fit. | 11 |
| Threshold values or cut-points | - Metric is based on categorizing an underlying continuous measure using threshold values or cut-points - This analytical approach should **only** be used for ‘class’ metrics - E.g., a catch-up growth ‘class’ is defined as $\geq$0.67 increase in HAZ, where the underlying continuous measure from which this class metric is derived is change in HAZ over a specified interval | 12 |
| Child-specific regression model – pre-defined structural model | - A regression model was built for each child, based on the repeated measures of size over time. - This code is used for models for which the shape of the curve has a pre-set defined functional form (e.g., exponential). - This code should **only** be used for analyses that involved classical parametric growth models (e.g., Jenss-Bayley) - Indicate the specific model in the next item | 13 |
| Child-specific regression model – empirical, data-driven model | - A regression model was built for each child, based on the repeated measures of size over time. - However, this code is used for all child-specific models other than those considered to be pre-defined structural models, e.g., those for which the parameters were selected based on their fit to the observed data (e.g., natural cubic regression spline). | 14 |
| Modeling of group(s) data - fixed effect regression with linear splines | - Regression of size parameter as a linear function of age/time using data from all children in a group - No random (child-specific) slopes - Metrics are limited to group averages (e.g., group average slope) | 15 |
| Modeling of group(s) data - fixed effect regression with non-linear curve | - Same as above, but the form of the regression function may be non-linear (e.g., cubic spline). - No random (child-specific) slopes - Metrics are limited to group averages (e.g., group average slope) | 16 |
| Modeling of group(s) data – random or mixed effect regression with or without linear splines | - Regression of size parameter as a function of age/time using data from all children in a group - Metrics are NOT limited to group averages, as child-specific metrics may be derived from child-specific random slopes | 17 |
| Modeling of group(s) data – random or mixed effect regression with non-linear functions | - Same as above, but the model incorporates non-linear terms such as cubic splines or polynomials. - Child-specific metrics may be derived from child-specific random slopes | 18 |
| Conditional regression | - Regression modeling in which size at one age is regressed on size at a previous time point, thereby generating a conditional metric of growth (the model residual). - The regression model includes data from multiple children (i.e., this is not a child-specific model). - May be used as a ‘step 1’ model to generate child-specific growth metrics for use in subsequent modeling of the association between growth and a later outcome. - Or, may be the primary analytical model in studies of predictors of (or risk factors for) growth; in such models, size at the previous time point may be one of several covariates. | 19 |
| SITAR model | - Shape-invariant non-linear mixed effects model of size as a function of age/time - Child-specific metrics may be derived from child-specific random effects: age-scaling factor, age intercepts and size intercepts. - Group average curve is also fitted. | 20 |
| Growth Mixture Modeling | - Structural equation modeling which identifies latent or unobservable subgroups within a given population - If the model is linear, then child-specific metrics may be derived from child-specific random slopes and/or intercepts. - If model is non-linear, child-specific metric is based on assignment to a ‘latent class’ or category of growth trajectories. | 21 |
| Latent Growth Curves | - Same as above, but variance and covariance are set to zero (i.e., all individual growth trajectories within a ‘latent class’ are assumed to be homogeneous) - Metrics are limited to random slope and/or intercept if the model is linear, and ‘latent class’ if the model is non-linear. | 22 |
| Machine Learning | - Model uses algorithms to learn patterns from the data without explicitly being programmed (i.e., hierarchical clustering, Bayesian modeling) - Child specific-metric is often but not limited to a class/category of growth trajectory, since machine learning is very flexible. | 23 |
| Other | - Select this code if author describes another analytical approach that has not yet been described above. | 24 |
