# Supplementary material for: Metrics of early childhood growth in recent epidemiological research: A scoping review
Source: PLoS One. 2018 Mar 20;13(3):e0194565. doi: 10.1371/journal.pone.0194565 (PMC5860780; doi:10.1371/journal.pone.0194565)
Supplement: S4 File — Studies included in the review. (DOCX) [file pone.0194565.s004.docx]

# **D. Studies included in the review**

| First author, year | Country | Sample size | Study design | Age at enrollment | Growth metric characteristics | | | | |
| --- | --- | --- | --- | --- | --- | --- | --- | --- | --- |
|  |  |  |  |  | Anthropometric parameter | Exposure or outcome | Content signature | Author-assigned label | Baseline adjustment^a^ |
| Abdelhalim,  2015 | Egypt | 29 | Cohort | Between 2 to 5 years of age | length | outcome | 22232312 | stunted linear growth | No/Not reported |
| Abdeyazdan, 2014 | Iran | 96 | Randomised controlled trial | At birth or within the first month of life | weight | outcome | 12121311 | gain | No/Not reported |
| Aris,  2015 | Singapore | 937 | Cohort | Prenatally | BMI | outcome | 22131417 | trajectory, velocity, change over time | No subsequent analyses |
|  |  |  |  |  | weight | outcome | 22131417 | trajectory, velocity, change over time | No subsequent analyses |
|  |  |  |  |  | length | outcome | 22131417 | trajectory, velocity, change over time | No subsequent analyses |
| Barkin,  2012 | USA | 75 | Randomised controlled trial | Between 2 to 5 years of age | BMI | outcome | 12121311 | growth pattern, change, change score | Yes, adjusted for initial size in all subsequent analyses |
| Batista,  2012 | Brazil | 1402 | Cohort | At birth or within the first month of life | length | outcome | 22222312 | catch-up growth, catch-down growth, growth, change | No/Not reported |
| Batra,  2016 | Guinea Bissau | 533 | Randomised controlled trial | Between 2 to 5 years of age | length | outcome | 22121311 | gain, change | Yes, adjusted for initial size in all subsequent analyses |
|  |  |  |  |  | weight | outcome | 22121311 | gain, change | Yes, adjusted for initial size in all subsequent analyses |
| Batscheider, 2014 | Germany | 3635 | Cohort | At birth or within the first month of life | BMI | exposure | 21232322 | trajectory pattern class, longitudinal growth, growth trajectory class, growth, velocity | No/Not reported |
| Belfort,  2011 | Australia | 613 | Randomised controlled trial | At birth or within the first month of life | weight | exposure | 22121417 | growth, growth rate, gain | Some analyses |
|  |  |  |  |  | length | exposure | 22121417 | growth, growth rate, linear growth | Some analyses |
|  |  |  |  |  | BMI | exposure | 22121417 | gain, growth, growth rate | Some analyses |
| Belfort,  2013 | USA | 941 | Cohort | At birth or within the first month of life | BMI | exposure | 22121311 | gain, change | Yes, adjusted for initial size in all subsequent analyses |
|  |  |  |  |  | length | exposure | 22121311 | linear growth, change | Yes, adjusted for initial size in all subsequent analyses |
| Belfort,  2016 | USA | 549 | Cohort | At birth or within the first month of life | weight | exposure | 12222312 | gain | Yes, adjusted for initial size in all subsequent analyses |
| Bisimwa,  201 | DRC | 1331 | Randomised controlled trial | Between 1 month and the first 2 years of age | length | outcome | 12121311 | growth, increment, gain | No/Not reported |
|  |  |  |  |  | weight | outcome | 12121311 | increment, gain, growth | No/Not reported |
|  |  |  |  |  | length | outcome | 22121311 | growth, change, gain | No/Not reported |
|  |  |  |  |  | weight | outcome | 22121311 | growth, change, gain | No/Not reported |
| Boguszewski, 2014 | 52 countries (Pfizer International Growth Database) | 620 | Cohort | Between 2 to 5 years of age | length | outcome | 22121819 | velocity | - |
| Bolisetty,  2014 | Australia | 145 | Cohort | At birth or within the first month of life | weight | outcome | 12121311 | gain | No subsequent analyses |
| Borschel,  2014 | USA | 40 | Cohort | Between 1 month and the first 2 years of age | length | outcome | 22121311 | improvement, gain | No subsequent analyses |
|  |  |  |  |  | weight | outcome | 22121311 | improvement, gain | No subsequent analyses |
|  |  |  |  |  | weight | outcome | 12121417 | gain | No subsequent analyses |
|  |  |  |  |  | length | outcome | 12121417 | gain | No subsequent analyses |
| Brunner,  2014 | Germany | 208 | Randomised controlled trial | Prenatally | weight | outcome | 12121311 | gain | Some analyses |
| Bruzzi,  2014 | Italy | 107 | Cohort | Between 1 month and the first 2 years of age | BMI | outcome | 22121311 | change | No subsequent analyses |
|  |  |  |  |  | length | outcome | 22121311 | change, catch-up growth | No subsequent analyses |
| Calarge,  2015 | USA | 73 | Randomised controlled trial | Between 2 to 5 years of age | BMI | outcome | 22121311 | change, gain | No/Not reported |
| Carter,  2013 | Canada | 1580 | Cohort | Between 1 month and the first 2 years of age | weight | exposure | 12222312 | gain | No subsequent analyses |
|  |  |  |  |  | BMI | outcome | 22131417 | rate of weight gain, growth, trend | Some analyses |
| Champion, 2010 | UK | 29 | Cohort | Between 1 month and the first 2 years of age | weight | outcome | 12121611 | change | No subsequent analyses |
| Cheng,  2016 | Singapore | 932 | Cohort | Prenatally | weight | outcome | 22121317 | longitudinal change, growth trajectory, gain, interval growth change | Yes, adjusted for initial size in all subsequent analyses |
|  |  |  |  |  | length | outcome | 22121317 | longitudinal change, growth trajectory, gain, interval growth change | Yes, adjusted for initial size in all subsequent analyses |
| Chivers,  2010 | Australia | 1330 | Cohort | Prenatally | BMI | outcome | 12131417 | trajectory | No/Not reported |
| Costet,  2015 | Guadeloupe | 197 | Cohort | Prenatally | length | outcome | 12131513 | growth velocity | No/Not reported |
|  |  |  |  |  | length | outcome | 12131713 | growth, linear growth | No/Not reported |
|  |  |  |  |  | weight | outcome | 12131713 | growth, linear growth | No/Not reported |
|  |  |  |  |  | weight | outcome | 12131513 | growth velocity | No/Not reported |
| Counts,  2013 | USA | 316 | Randomised controlled trial | Between 2 to 5 years of age | length | outcome | 22121311 | change | No/Not reported |
|  |  |  |  |  | length | outcome | 22122011 | time to reach | No/Not reported |
| Counts,  2015 | USA | 316 | Randomised controlled trial | Between 2 to 5 years of age | length | outcome | 22121311 | gain, deficit | No/Not reported |
| Crespo,  2012 | USA | 808 | Randomised controlled trial | Between 2 to 5 years of age | BMI | outcome | 22131417 | change | Yes, adjusted for initial size in all subsequent analyses |
| Czechowicz, 2015 | USA | 76 | Cohort | Between 1 month and the first 2 years of age | BMI | outcome | 22121311 | growth, change, gain | No/Not reported |
| Degorre,  2015 | France | 38 | Randomised controlled trial | At birth or within the first month of life | weight | outcome | 12121311 | change, gain | No subsequent analyses |
| Downs,  2014 | Australia | 229 | Cohort | At birth or within the first month of life | weight | outcome | 22121311 | change | No/Not reported |
| Dupont,  2014 | Belgium, France | 75 | Randomised controlled trial | Between 1 month and the first 2 years of age | weight | outcome | 22121311 | change, catch-up growth, growth, gain | No subsequent analyses |
|  |  |  |  |  | length | outcome | 22121311 | change, growth | No subsequent analyses |
|  |  |  |  |  | BMI | outcome | 22121311 | change, growth, gain | No subsequent analyses |
| Ernst,  2012 | Netherlands | 187 | Cohort | Between 2 to 5 years of age | length | outcome | 22121311 | difference, change | No/Not reported |
| Escribano,  2012 | Europe | 66 | Randomised controlled trial | At birth or within the first month of life | weight | exposure | 12121411 | gain velocity | Some analyses |
|  |  |  |  |  | length | exposure | 12121411 | gain velocity | Some analyses |
| Fabiansen,  2016 | Burkina Faso | 468 | Cohort | Between 1 month and the first 2 years of age | weight | outcome | 12121611 | change, percent change | No subsequent analyses |
|  |  |  |  |  | weight | outcome | 12121717 | gain velocity | No subsequent analyses |
| Feigerlova, 2010 | France | 624 | Cohort | Between 2 to 5 years of age | BMI | both | 22122224 | growth velocity | No subsequent analyses |
|  |  |  |  |  | BMI | outcome | 22121311 | change | Some analyses |
| Fergusson,  2014 | New Zealand | 1390 | Cohort | At birth or within the first month of life | weight | exposure | 12222312 | growth | No/Not reported |
| Franchetti,  2014 | Japan | 45392 | Cohort | At birth or within the first month of life | BMI | outcome | 21232321 | growth pattern, trajectory | No subsequent analyses |
|  |  |  |  |  | BMI | outcome | 11131415 | growth | Yes, adjusted for initial size in all subsequent analyses |
| Fahraeus,  2012 | Sweden | 496 | Cohort | At birth or within the first month of life | weight | exposure | 12121611 | relative gain, gain | No/Not reported |
| Gerards,  2015 | Netherlands | 86 | Randomised controlled trial | Between 2 to 5 years of age | BMI | outcome | 21131315 | gain, change | No/Not reported |
| Gerber,  2016 | USA | 38522 | Cohort | At birth or within the first month of life | weight | outcome | 12131418 | growth trajectory, trajectory, rate of gain, weight change | Yes, adjusted for initial size in all subsequent analyses |
| Gianni,  2014 | Italy | 130 | Prospective, non randomized interventional cohort | At birth or within the first month of life | weight | outcome | 12121711 | growth, gain velocity | Yes, adjusted for initial size in all subsequent analyses |
| Gilbert-Diamond,  2010 | Colombia | 479 | Cohort | Between 2 to 5 years of age | length | outcome | 12131417 | change, linear growth | Some analyses |
|  |  |  |  |  | BMI | outcome | 12131417 | change | Some analyses |
| Gutknecht, 2015 | USA | 363 | Cohort | Between 2 to 5 years of age | BMI | outcome | 22131417 | gain, change | No/Not reported |
| Guyer,  2012 | Switzerland | 37 | Randomised controlled trial | At birth or within the first month of life | weight | outcome | 12121411 | gain | No/Not reported |
| Harvey,  2012 | UK | 628 | Cohort | Prenatally | length | both | 22121819 | conditional change, growth, growth trajectory, velocity, linear growth | - |
| Heney,  2015 | USA | 181 | Retrospective chart review | Between 2 to 5 years of age | BMI | outcome | 22121311 | change | No/Not reported |
| Hernandez, 2011 | USA | 1140 | Cohort | Between 2 to 5 years of age | BMI | outcome | 12131417 | trajectory, rate of change | No subsequent analyses |
| Howe,  2012 | UK | 12366 | Cohort | Prenatally | length | outcome | 12121417 | trajectory, growth rate, growth trajectory | No subsequent analyses |
| Imai,  2014 | Iceland | 154 | Cohort | At birth or within the first month of life | weight | outcome | 12121311 | change, gain | Yes, adjusted for initial size in all subsequent analyses |
| Jacquot,  2011 | France | 29 | Cohort | Prenatally | weight | outcome | 12121311 | gain | No/Not reported |
| Johnson,  2010 | Romania | 110 | Randomised controlled trial | Between 1 month and the first 2 years of age | length | outcome | 12131417 | growth rate | No subsequent analyses |
|  |  |  |  |  | length | both | 22121311 | growth | Some analyses |
|  |  |  |  |  | weight | outcome | 12131417 | growth rate | No subsequent analyses |
|  |  |  |  |  | weight | both | 22121311 | growth | Some analyses |
| Johnson,  2012 | UK | 2181 | Cohort | At birth or within the first month of life | BMI | exposure | 22131313 | change, gain, growth pattern | No/Not reported |
|  |  |  |  |  | length | exposure | 22131313 | growth, growth pattern | No/Not reported |
|  |  |  |  |  | weight | exposure | 22131313 | growth pattern, growth, change, gain | No/Not reported |
| Johnston,  2015 | USA | 353 | Randomised controlled trial | At birth or within the first month of life | weight | outcome | 12121414 | growth rate | No/Not reported |
|  |  |  |  |  | length | outcome | 12121414 | growth rate | No/Not reported |
| Joseph,  2015 | Peru | 1760 | Randomised controlled trial | Between 1 month and the first 2 years of age | weight | outcome | 22121311 | change | No subsequent analyses |
|  |  |  |  |  | weight | outcome | 12121311 | gain | No subsequent analyses |
|  |  |  |  |  | length | outcome | 12121311 | gain | No subsequent analyses |
|  |  |  |  |  | length | outcome | 22121311 | change | No subsequent analyses |
| Jourdan,  2012 | France | 39 | Cohort | At birth or within the first month of life | BMI | outcome | 22131417 | rate of change, rate of gain, gain | No/Not reported |
| Khan,  2013 | Bangladesh | 2168 | Randomised controlled trial | Prenatally | weight | outcome | 22131315 | growth | Some analyses |
|  |  |  |  |  | length | outcome | 22131315 | growth, linear growth, linear growth trajectory | Some analyses |
| Kinlin,  2012 | Canada | 226 | Cohort | Between 1 month and the first 2 years of age | weight | outcome | 12121611 | gain, percent gain | No/Not reported |
| Kong,  2015 | USA | 57 | Cohort | Between 1 month and the first 2 years of age | weight | exposure | 12121411 | gain | Yes, adjusted for initial size in all subsequent analyses |
| Lauhkonen,  2015 | Finland | 99 | Cohort | At birth or within the first month of life | weight | outcome | 12222312 | gain | Yes, adjusted for initial size in all subsequent analyses |
| Lima,  2014 | Brazil | 570 | Cohort | At birth or within the first month of life | weight | outcome | 22121411 | gain, evolution | No/Not reported |
| Lin,  2015a | Singapore | 976 | Cohort | Prenatally | weight | outcome | 11131615 | percentage change | No/Not reported |
|  |  |  |  |  | length | outcome | 11131615 | percentage change | No/Not reported |
| Lin,  2015b | USA, China | 1714 | Cohort | At birth or within the first month of life | weight | both | 22121311 | change | Yes, adjusted for initial size in all subsequent analyses |
|  |  |  |  |  | weight | outcome | 22122411 | performance gap | No subsequent analyses |
| Lindberg,  2015 | Sweden | 380 | Cohort | Prenatally | weight | exposure | 22121311 | growth velocity, change | No/Not reported |
|  |  |  |  |  | length | exposure | 22121311 | change | No/Not reported |
| Linglart,  2011 | France | 61 | Cohort | Between 1 month and the first 2 years of age | length | outcome | 22121311 | change, gain | Some analyses |
| Loomis,  2014 | USA | 253 | Retrospective chart review | At birth or within the first month of life | weight | outcome | 22121311 | change | No subsequent analyses |
| Lundeen,  2014 | Brazil, Guatemala, India, Philippines, South Africa | 5287 | Cohort | At birth or within the first month of life | length | outcome | 22222312 | recovery from stunting | No subsequent analyses |
| Maas,  2013 | Germany | 224 | Cohort | At birth or within the first month of life | weight | outcome | 22121311 | difference | No subsequent analyses |
|  |  |  |  |  | weight | outcome | 12121411 | gain | No subsequent analyses |
| Magnus,  2015 | Norway, Denmark | 99832 | Cohort | Prenatally | weight | exposure | 12121311 | change, growth | Some analyses |
|  |  |  |  |  | length | exposure | 12121311 | change, growth | Some analyses |
|  |  |  |  |  | weight | exposure | 22121311 | change, growth | Some analyses |
|  |  |  |  |  | length | exposure | 22121311 | change, growth | Some analyses |
| Maleta,  2015 | Malawi | 1411 | Randomised controlled trial | Between 1 month and the first 2 years of age | length | outcome | 12121311 | change | Some analyses |
|  |  |  |  |  | length | outcome | 22121311 | change | Some analyses |
|  |  |  |  |  | weight | outcome | 22121311 | change | Some analyses |
|  |  |  |  |  | weight | outcome | 12121311 | change | Some analyses |
| Martins,  2013 | Portugal | 49 | Cohort | Between 1 month and the first 2 years of age | length | outcome | 21232322 | trajectory, patterns of change over time | Some analyses |
|  |  |  |  |  | weight | outcome | 21232322 | trajectory, patterns of change over time | Some analyses |
| Matijasevich, 2012 | Brazil | 4053 | Cohort | Prenatally | length | outcome | 12131417 | growth velocity, growth, growth rates, growth trajectory | No/Not reported |
| Mazaki-Tovi, 2011 | Israel | 68 | Cohort | At birth or within the first month of life | weight | outcome | 12121311 | gain | No subsequent analyses |
| McCloskey, 2016 | Australia | 835 | Cohort | Prenatally | weight | exposure | 12121311 | gain | Yes, adjusted for initial size in all subsequent analyses |
| McLeod,  2016 | Australia | 40 | Randomised controlled trial | At birth or within the first month of life | weight | outcome | 12131617 | gain velocity | No/Not reported |
| Minich,  2010 | USA | 60 | Cohort | Between 1 month and the first 2 years of age | weight | outcome | 22121311 | gain, change | No/Not reported |
| Mitter,  2012 | Brazil | 213 | Randomised controlled trial | Between 1 month and the first 2 years of age | weight | outcome | 22121311 | growth, gain | No/Not reported |
|  |  |  |  |  | length | outcome | 22121311 | growth, gain | No/Not reported |
| Monse,  2012 | Philippines | 164 | Randomised controlled trial | Between 2 to 5 years of age | weight | outcome | 12121311 | growth, change, difference | No/Not reported |
|  |  |  |  |  | weight | outcome | 22121819 | conditional velocity, conditional growth velocity | - |
|  |  |  |  |  | length | outcome | 22121819 | conditional velocity, conditional growth velocity | - |
|  |  |  |  |  | length | outcome | 12121311 | growth, change, difference | No/Not reported |
|  |  |  |  |  | length | outcome | 22121311 | growth, change, difference | No/Not reported |
|  |  |  |  |  | weight | outcome | 22121311 | growth, change, difference | No/Not reported |
|  |  |  |  |  | BMI | outcome | 22121819 | conditional velocity, conditional growth velocity | - |
|  |  |  |  |  | BMI | outcome | 22121311 | change, difference | No/Not reported |
|  |  |  |  |  | BMI | outcome | 12121311 | change, difference | No/Not reported |
| Nackers,  2010 | Niger | 421 | Randomised controlled trial | Between 1 month and the first 2 years of age | length | outcome | 12121411 | gain | No subsequent analyses |
|  |  |  |  |  | length | outcome | 22121411 | gain | No subsequent analyses |
|  |  |  |  |  | weight | outcome | 12121711 | gain | No subsequent analyses |
| Natarajan,  2014 | USA | 375 | Cohort | At birth or within the first month of life | weight | outcome | 12121411 | gain | No/Not reported |
| Navardauskaite, 2014 | Lithuania | 67 | Cohort | At birth or within the first month of life | length | outcome | 12121411 | velocity, growth velocity, growth | No/Not reported |
| Parra-Medina, 2015 | USA | 118 | Randomised controlled trial | Between 2 to 5 years of age | weight | outcome | 12132312 | change, weight gain | No/Not reported |
|  |  |  |  |  | BMI | outcome | 22132312 | change, weight gain | No/Not reported |
| Peiler,  2014 | Germany | 52 | Cohort | At birth or within the first month of life | length | outcome | 22121311 | growth, change | No/Not reported |
|  |  |  |  |  | weight | outcome | 12121711 | fractional growth rate, growth, growth velocity | No/Not reported |
|  |  |  |  |  | BMI | outcome | 22121311 | growth, change | No/Not reported |
|  |  |  |  |  | weight | outcome | 22121311 | growth, change | No/Not reported |
| Perng,  2016 | USA | 957 | Cohort | Prenatally | BMI | exposure | 22121311 | gain, change | Some analyses |
|  |  |  |  |  | length | exposure | 22121311 | change, gain, linear growth | Some analyses |
| Pimpin,  2016 | UK | 2154 | Cohort | Between 1 month and the first 2 years of age | weight | both | 12131417 | rate of growth, gain | Some analyses |
|  |  |  |  |  | BMI | both | 12131417 | rate of growth | Yes, adjusted for initial size in all subsequent analyses |
|  |  |  |  |  | length | both | 12131417 | rate of growth | Yes, adjusted for initial size in all subsequent analyses |
| Radhakrishna, 2010 | India | 80 | Cohort | Between 1 month and the first 2 years of age | weight | outcome | 12121711 | gain | No/Not reported |
| Ram Kumar, 2012 | India | 63 | Randomised controlled trial | At birth or within the first month of life | length | outcome | 12121411 | growth, linear growth velocity | No/Not reported |
| Ramel,  2011 | USA | 123 | Cohort | At birth or within the first month of life | length | outcome | 12121415 | growth, change | Some analyses |
|  |  |  |  |  | weight | outcome | 12121415 | growth, change | Yes, adjusted for initial size in all subsequent analyses |
| Ranke,  2012a | Germany | 210 | Cohort | Between 2 to 5 years of age | length | both | 12121411 | velocity | No/Not reported |
|  |  |  |  |  | length | both | 22121311 | change | No/Not reported |
| Ranke,  2012b | 52 countries (Pfizer International Growth Database) | 1279 | Cohort | Between 2 to 5 years of age | length | exposure | 12131411 | velocity | No/Not reported |
|  |  |  |  |  | length | exposure | 22121311 | change | No/Not reported |
|  |  |  |  |  | weight | exposure | 22121311 | gain | No/Not reported |
| Reeske,  2013 | Germany | 1287 | Cohort | Between 2 to 5 years of age | weight | outcome | 22222312 | gain | Some analyses |
|  |  |  |  |  | weight | outcome | 22121311 | change, growth, gain | Some analyses |
| Rogers,  2014 | UK | 31 | Cohort | At birth or within the first month of life | weight | outcome | 22121311 | gain | No/Not reported |
| Saari,  2015 | Finland | 177 | Cohort | At birth or within the first month of life | length | outcome | 22121311 | change, growth | No/Not reported |
|  |  |  |  |  | BMI | outcome | 22121311 | change, growth | No/Not reported |
| Saleem,  2014 | Pakistan | 194 | Randomised controlled trial | Between 1 month and the first 2 years of age | weight | outcome | 12131417 | linear growth | No/Not reported |
|  |  |  |  |  | length | outcome | 12131417 | linear growth | No/Not reported |
| Sanders,  2015 | USA | 6805 | Cohort | Between 1 month and the first 2 years of age | weight | exposure | 22232312 | growth trajectory category | No subsequent analyses |
|  |  |  |  |  | BMI | exposure | 22232312 | growth trajectory category | No/Not reported |
|  |  |  |  |  | length | exposure | 22232312 | growth trajectory category | No subsequent analyses |
| Schreiner,  2010 | Germany | 51 | Cohort | At birth or within the first month of life | length | both | 22122411 | length corrected for target height | No/Not reported |
| Silventoinen, 2014 | Japan | 740 | Cohort | At birth or within the first month of life | length | exposure | 12131417 | growth, growth trajectory, linear growth | No/Not reported |
|  |  |  |  |  | weight | exposure | 12131417 | growth, growth trajectory, linear growth | No/Not reported |
| Skoner,  201 | USA | 184 | Randomised controlled trial | Between 2 to 5 years of age | length | outcome | 12131417 | growth velocity | No subsequent analyses |
|  |  |  |  |  | length | outcome | 12131414 | growth velocity | No subsequent analyses |
| Spiegler,  2016 | Germany | 1433 | Cohort | Prenatally | weight | outcome | 22121311 | growth rate, change | No/Not reported |
| Stark,  2014 | USA | 33 | Randomised controlled trial | Between 2 to 5 years of age | BMI | outcome | 22131317 | gain, change | Some analyses |
| Surkan,  2014 | USA | 6550 | Cohort | Between 1 month and the first 2 years of age | BMI | outcome | 12131417 | growth trajectory | Some analyses |
|  |  |  |  |  | length | outcome | 12131417 | growth trajectory | Some analyses |
| Suzuki,  2011 | Japan | 1603 | Cohort | Prenatally | BMI | outcome | 21131415 | trajectory, growth, growth trajectory | No/Not reported |
| Tandon,  2012 | India | 565 | Cohort | At birth or within the first month of life | BMI | exposure | 22121819 | growth, change, conditional growth | - |
|  |  |  |  |  | length | exposure | 22121819 | growth, conditional change, gain, conditional growth | - |
| Taylor,  2012 | France | 176 | Cohort | Between 1 month and the first 2 years of age | BMI | outcome | 22222312 | progression | No/Not reported |
| Thakwalakwa, 2010 | Malawi | 188 | Randomised controlled trial | Between 1 month and the first 2 years of age | weight | outcome | 12121311 | change, gain | No subsequent analyses |
|  |  |  |  |  | length | outcome | 12121311 | change | No subsequent analyses |
|  |  |  |  |  | length | outcome | 22121311 | change | No subsequent analyses |
|  |  |  |  |  | weight | outcome | 22121311 | change, gain | No subsequent analyses |
| Thakwalakwa, 2012 | Malawi | 299 | Randomised controlled trial | Between 1 month and the first 2 years of age | length | outcome | 12121311 | gain | No subsequent analyses |
|  |  |  |  |  | weight | outcome | 12121311 | gain | No subsequent analyses |
|  |  |  |  |  | weight | outcome | 22121311 | change | No subsequent analyses |
|  |  |  |  |  | length | outcome | 22121311 | change | No subsequent analyses |
| Thearle,  2015 | USA | 1920 | Cohort | At birth or within the first month of life | BMI | both | 11232322 | class | Some analyses |
|  |  |  |  |  | BMI | both | 21232322 | pattern of change, trajectory class, trajectory group | Some analyses |
| Thomas,  2012 | India | 61 | Randomised controlled trial | At birth or within the first month of life | weight | outcome | 12121711 | gain | No subsequent analyses |
| Trehan,  2013 | Malawi | 2767 | Randomised controlled trial | Between 1 month and the first 2 years of age | weight | outcome | 12121711 | gain | No subsequent analyses |
|  |  |  |  |  | length | outcome | 12121411 | gain | No subsequent analyses |
| Tu,  2015 | Canada | 7253 | Cohort | Between 1 month and the first 2 years of age | BMI | outcome | 11232321 | trajectory | Yes, adjusted for initial size in all subsequent analyses |
| Vieira,  2014 | Brazil | 257 | Cohort | Between 2 to 5 years of age | weight | exposure | 12121411 | gain rate | Some analyses |
| Wasniewska, 2013 | Italy | 25 | Cohort | Between 2 to 5 years of age | length | outcome | 22121311 | velocity, catch-up growth, growth, change | No/Not reported |
| Wijlaars,  2011 | UK | 2081 | Cohort | At birth or within the first month of life | weight | outcome | 22121311 | change, growth | No/Not reported |
|  |  |  |  |  | weight | outcome | 22222312 | growth | No/Not reported |
| Wright,  2015 | UK | 30 | Retrospective chart review | Between 1 month and the first 2 years of age | length | outcome | 22222312 | growth pattern, change | No subsequent analyses |
|  |  |  |  |  | BMI | outcome | 22222312 | growth pattern, change | No subsequent analyses |
|  |  |  |  |  | weight | outcome | 22121311 | growth pattern, gain, change | No subsequent analyses |
| Yanagida,  2015 | Japan | 195 | Cohort | Between 1 month and the first 2 years of age | length | outcome | 22121311 | growth, change, rate | No subsequent analyses |
| Yang,  2014 | USA | 36 | Randomised controlled trial | At birth or within the first month of life | weight | outcome | 12121411 | growth rate, gain | No/Not reported |
|  |  |  |  |  | length | outcome | 12121411 | growth rate, gain | No/Not reported |
| Zhao,  2015 | China | 3012 | Cohort | At birth or within the first month of life | weight | outcome | 12121411 | growth rate, trajectory | Some analyses |
|  |  |  |  |  | length | outcome | 12121411 | growth rate, trajectory | Some analyses |
| Zheng,  2015 | Denmark | 352 | Randomised controlled trial | Between 2 to 5 years of age | BMI | outcome | 22121311 | change, gain, delta | Yes, adjusted for initial size in all subsequent analyses |
|  |  |  |  |  | weight | outcome | 12121311 | change, gain, delta | No/Not reported |
| Zung,  2012 | Israel | 55 | Cohort | Between 2 to 5 years of age | length | outcome | 22122224 | velocity | No/Not reported |
|  |  |  |  |  | weight | outcome | 22121311 | gain | No/Not reported |
|  |  |  |  |  | BMI | outcome | 22121311 | gain | No/Not reported |
|  |  |  |  |  | BMI | outcome | 12121311 | gain | No/Not reported |
| de Hoog,  2011 | Netherlands | 3156 | Cohort | Prenatally | weight | exposure | 12121411 | gain | Yes, adjusted for initial size in all subsequent analyses |
| de Ruyter,  2012 | Netherlands | 477 | Randomised controlled trial | Between 2 to 5 years of age | length | outcome | 22121311 | change, gain | No/Not reported |
|  |  |  |  |  | weight | outcome | 12121311 | change, gain | No/Not reported |
|  |  |  |  |  | BMI | outcome | 22121311 | change | No/Not reported |
|  |  |  |  |  | length | outcome | 12121311 | change, gain | No/Not reported |
| ud Din,  2013 | UK | 11499 | Cohort | Prenatally | weight | exposure | 22121819 | conditional gain, velocity | - |
|  |  |  |  |  | length | exposure | 22121819 | conditional gain, velocity | - |
| van Gool,  2010 | Netherlands | 28 | Cohort | Between 2 to 5 years of age | length | outcome | 22121311 | gain | No subsequent analyses |
| van der Gugten, 2012 | Netherlands | 235 | Cohort | At birth or within the first month of life | weight | exposure | 22222312 | gain, gain pattern, growth pattern | No/Not reported |
|  |  |  |  |  | weight | exposure | 22121311 | gain | No/Not reported |
| van der Willik, 2015 | Netherlands | 3367 | Cohort | Prenatally | weight | exposure | 22222312 | gain | No/Not reported |

^a^ Not applicable to conditional growth metrics

# **Reference citations for studies included in the scoping review**

| Abdelhalim A, Elshal AM, Elsawy AA, Helmy TE, Orban HA, Dawaba ME, Hafez AT. Bricker conduit for pediatric urinary diversion—should we still offer it?. The Journal of urology. 2015 Nov 30;194(5):1414-9. |
| --- |
| Abdeyazdan Z, Ghasemi S, Marofi M, Berjis N. Motor Responses and Weight Gaining in Neonates through Use of Two Methods of Earmuff and Receiving Silence in NICU. The Scientific World Journal. 2014 Dec 30;2014. |
| Aris IM, Soh SE, Tint MT, Saw SM, Rajadurai VS, Godfrey KM, Gluckman PD, Yap F, Chong YS, Lee YS. Associations of gestational glycemia and prepregnancy adiposity with offspring growth and adiposity in an Asian population. The American journal of clinical nutrition. 2015 Nov 1;102(5):1104-12. |
| Barkin SL, Gesell SB, Po’e EK, Escarfuller J, Tempesti T. Culturally tailored, family-centered, behavioral obesity intervention for Latino-American preschool-aged children. Pediatrics. 2012 Sep 1;130(3):445-56. |
| Batista RF, Silva AA, Barbieri MA, Simões VM, Bettiol H. Factors associated with height catch-up and catch-down growth among schoolchildren. PloS one. 2012 Mar 12;7(3):e32903. |
| Batra P, Schlossman N, Balan I, Pruzensky W, Balan A, Brown C, Gamache MG, Schleicher MM, de Sa AB, Saltzman E, Wood L. A Randomized Controlled Trial Offering Higher-Compared with Lower-Dairy Second Meals Daily in Preschools in Guinea-Bissau Demonstrates an Attendance-Dependent Increase in Weight Gain for Both Meal Types and an Increase in Mid-Upper Arm Circumference for the Higher-Dairy Meal. The Journal of nutrition. 2016 Jan 1;146(1):124-32. |
| Batscheider A, Rzehak P, Teuner CM, Wolfenstetter SB, Leidl R, von Berg A, Berdel D, Hoffmann B, Heinrich J. Development of BMI values of German children and their healthcare costs. Economics & Human Biology. 2014 Jan 31;12:56-66. |
| Belfort MB, Rifas-Shiman SL, Sullivan T, Collins CT, McPhee AJ, Ryan P, Kleinman KP, Gillman MW, Gibson RA, Makrides M. Infant growth before and after term: effects on neurodevelopment in preterm infants. Pediatrics. 2011 Sep 20:peds-2011. |
| Belfort MB, Gillman MW, Buka SL, Casey PH, McCormick MC. Preterm infant linear growth and adiposity gain: trade-offs for later weight status and intelligence quotient. The Journal of pediatrics. 2013 Dec 31;163(6):1564-9. |
| Belfort MB, Kuban KC, O'Shea TM, Allred EN, Ehrenkranz RA, Engelke SC, Leviton A, Extremely Low Gestational Age Newborn (ELGAN) Study Investigators. Weight Status in the First 2 Years of Life and Neurodevelopmental Impairment in Extremely Low Gestational Age Newborns. The Journal of pediatrics. 2016 Jan 31;168:30-5. |
| Bisimwa G, Owino VO, Bahwere P, Dramaix M, Donnen P, Dibari F, Collins S. Randomized controlled trial of the effectiveness of a soybean-maize-sorghum–based ready-to-use complementary food paste on infant growth in South Kivu, Democratic Republic of Congo. The American journal of clinical nutrition. 2012 May 1;95(5):1157-64. |
| Boguszewski MC, Lindberg A, Wollmann HA. Three-Year Growth Response to Growth Hormone Treatment in Very Young Children Born Small for Gestational Age—Data from KIGS. The Journal of Clinical Endocrinology & Metabolism. 2014 Apr 23;99(8):2683-8. |
| Bolisetty S, Pharande P, Nirthanakumaran L, Do TQ, Osborn D, Smyth J, Sinn J, Lui K. Improved nutrient intake following implementation of the consensus standardised parenteral nutrition formulations in preterm neonates–a before-after intervention study. BMC pediatrics. 2014 Dec 17;14(1):1. |
| Borschel MW, Antonson DL, Murray ND, Oliva-Hemker M, Mattis LE, Kerzner B, Tolia V, Baggs G. Two single group, prospective, baseline-controlled feeding studies in infants and children with chronic diarrhea fed a hypoallergenic free amino acid-based formula. BMC pediatrics. 2014 May 29;14(1):1. |
| Brunner S, Schmid D, Hüttinger K, Much D, Brüderl M, Sedlmeier EM, Kratzsch J, Amann‐Gassner U, Bader BL, Hauner H. Effect of reducing the n‐6/n‐3 fatty acid ratio on the maternal and fetal leptin axis in relation to infant body composition. Obesity. 2014 Jan 1;22(1):217-24. |
| Bruzzi P, Predieri B, Corrias A, Marsciani A, Street ME, Rossidivita A, Paolucci P, Iughetti L. Final height and body mass index in adult survivors of childhood acute lymphoblastic leukemia treated without cranial radiotherapy: a retrospective longitudinal multicenter Italian study. BMC pediatrics. 2014 Sep 22;14(1):1. |
| Calarge CA, Ziegler EE, Del Castillo N, Aman M, McDougle CJ, Scahill L, McCracken JT, Arnold LE. Iron homeostasis during risperidone treatment in children and adolescents. The Journal of clinical psychiatry. 2015 Aug 4;76(11):1500-5. |
| Carter MA, Dubois L, Tremblay MS, Taljaard M. The influence of place on weight gain during early childhood: a population-based, longitudinal study. Journal of Urban Health. 2013 Apr 1;90(2):224-39. |
| Champion H, Ramaswami U, Imrie J, Lachmann RH, Gallagher J, Cox TM, Wraith JE. Dietary modifications in patients receiving miglustat. Journal of inherited metabolic disease. 2010 Dec 1;33(3):379-83. |
| Cheng TS, Loy SL, Cheung YB, Chan JK, Pang WW, Godfrey KM, Gluckman PD, Kwek K, Saw SM, Chong YS, Lee YS. Sexually dimorphic response to feeding mode in the growth of infants. The American journal of clinical nutrition. 2016 Feb 1;103(2):398-405. |
| Chivers P, Hands B, Parker H, Bulsara M, Beilin LJ, Kendall GE, Oddy WH. Body mass index, adiposity rebound and early feeding in a longitudinal cohort (Raine Study). International Journal of Obesity. 2010 Jul 1;34(7):1169-76. |
| Costet N, Pelé F, Comets E, Rouget F, Monfort C, Bodeau-Livinec F, Linganiza EM, Bataille H, Kadhel P, Multigner L, Cordier S. Perinatal exposure to chlordecone and infant growth. Environmental research. 2015 Oct 31;142:123-34. |
| Counts DR, Silverman LA, Geffner ME, Rajicic N, Hey-Hadavi J, Thornton PS, Wajnrajch MP, ISS Study Group. A four-year, open-label, multi-center, randomized, two-arm study of Genotropin® in patients with idiopathic short stature: comparison of an individualized, target-driven treatment regimen to standard dosing of Genotropin®-analysis of two-year data. Hormone Research in Paediatrics. 2013 Sep 10;80(4):242-51. |
| Counts DR, Silverman LA, Rajicic N, Geffner ME, Newfield RS, Thornton P, Carakushansky M, Escobar O, Rapaport R, Levitsky L, Rotenstein D. A 4-Year, Open-Label, Multicenter, Randomized Trial of Genotropin® Growth Hormone in Patients with Idiopathic Short Stature: Analysis of 4-Year Data Comparing Efficacy, Efficiency, and Safety between an Individualized, Target-Driven Regimen and Standard Dosing. Hormone Research in Paediatrics. 2015 May 1;84(2):79-87. |
| Crespo NC, Elder JP, Ayala GX, Slymen DJ, Campbell NR, Sallis JF, McKenzie TL, Baquero B, Arredondo EM. Results of a multi-level intervention to prevent and control childhood obesity among Latino children: the Aventuras Para Niños Study. Annals of Behavioral Medicine. 2012 Feb 1;43(1):84-100. |
| Czechowicz JA, Chang KW. Catch-up growth in infants with laryngomalacia after supraglottoplasty. International journal of pediatric otorhinolaryngology. 2015 Aug 31;79(8):1333-6. |
| Degorre C, Décima P, Dégrugilliers L, Ghyselen L, Bach V, Libert JP, Tourneux P. A mean body temperature of 37° C for incubated preterm infants is associated with lower energy costs in the first 11 days of life. Acta Paediatrica. 2015 Jun 1;104(6):581-8. |
| Downs J, Wong K, Ravikumara M, Ellaway C, Elliott EJ, Christodoulou J, Jacoby P, Leonard H. Experience of gastrostomy using a quality care framework: the example of Rett syndrome. Medicine. 2014 Dec;93(28). |
| Dupont C, Kalach N, Soulaines P, Bradatan E, Lachaux A, Payot F, de Blay F, Guénard-Bilbault L, Hatahet R, Mulier S. A Thickened Amino-Acid Formula in Infants with Cow’s Milk Allergy Failing to Respond to Protein Hydrolysate Formulas: A Randomized Double-Blind Trial. Pediatric Drugs. 2014 Dec 1;16(6):513-22. |
| Ernst MA, Simons MY, Gerver AJ, Zandwijken GR, Zimmermann LJ, Gerver WJ. Change in total body water as a predictive tool for growth hormone treatment response. Hormone Research in Paediatrics. 2012 Jul 18;78(1):18-23. |
| Escribano J, Luque V, Ferre N, Mendez-Riera G, Koletzko B, Grote V, Demmelmair H, Bluck L, Wright A, Closa-Monasterolo R. Effect of protein intake and weight gain velocity on body fat mass at 6 months of age: the EU Childhood Obesity Programme. International journal of obesity. 2012 Apr 1;36(4):548-53. |
| Fabiansen C, Phelan KP, Cichon B, Ritz C, Briend A, Michaelsen KF, Friis H, Shepherd S. Short children with a low midupper arm circumference respond to food supplementation: an observational study from Burkina Faso. The American journal of clinical nutrition. 2016 Feb 1;103(2):415-21. |
| Feigerlova E, Diene G, Oliver I, Gennero I, Salles JP, Arnaud C, Tauber M. Elevated insulin-like growth factor-I values in children with Prader-Willi syndrome compared with growth hormone (GH) deficiency children over two years of GH treatment. The Journal of Clinical Endocrinology & Metabolism. 2010 Oct;95(10):4600-8. |
| Fergusson DM, McLeod GF, Horwood LJ. Breast feeding, infant growth, and body mass index at 30 and 35 years. Paediatric and perinatal epidemiology. 2014 Nov 1;28(6):545-52. |
| Franchetti Y, Ide H. Socio-demographic and lifestyle factors for child’s physical growth and adiposity rebound of Japanese children: a longitudinal study of the 21st century longitudinal survey in newborns. BMC public health. 2014 Apr 9;14(1):1. |
| Fåhraeus C, Wendt LK, Nilsson M, Isaksson H, Alm A, Andersson‐Gäre B. Overweight and obesity in twenty‐year‐old Swedes in relation to birthweight and weight development during childhood. Acta Paediatrica. 2012 Jun 1;101(6):637-42. |
| Gerards SM, Dagnelie PC, Gubbels JS, Van Buuren S, Hamers FJ, Jansen MW, Van Der Goot OH, De Vries NK, Sanders MR, Kremers SP. The effectiveness of Lifestyle Triple P in the Netherlands: a randomized controlled trial. PloS one. 2015 Apr 7;10(4):e0122240. |
| Gerber JS, Bryan M, Ross RK, Daymont C, Parks EP, Localio AR, Grundmeier RW, Stallings VA, Zaoutis TE. Antibiotic Exposure During the First 6 Months of Life and Weight Gain During Childhood. JAMA. 2016 Mar 22;315(12):1258-65. |
| Giannì ML, Roggero P, Colnaghi MR, Piemontese P, Amato O, Orsi A, Morlacchi L, Mosca F. The role of nutrition in promoting growth in pre-term infants with bronchopulmonary dysplasia: a prospective non-randomised interventional cohort study. BMC pediatrics. 2014 Sep 22;14(1):1. |
| Gilbert-Diamond D, Baylin A, Mora-Plazas M, Marin C, Arsenault JE, Hughes MD, Willett WC, Villamor E. Vitamin D deficiency and anthropometric indicators of adiposity in school-age children: a prospective study. The American journal of clinical nutrition. 2010 Dec 1;92(6):1446-51. |
| Gutknecht SM, Schwartz MH, Munger ME. Ambulatory children with cerebral palsy do not exhibit unhealthy weight gain following selective dorsal rhizotomy. Developmental Medicine & Child Neurology. 2015 Nov 1;57(11):1070-5. |
| Guyer C, Huber R, Fontijn J, Bucher HU, Nicolai H, Werner H, Molinari L, Latal B, Jenni OG. Cycled light exposure reduces fussing and crying in very preterm infants. Pediatrics. 2012 Jul 1;130(1):e145-51. |
| Harvey NC, Mahon PA, Kim M, Cole ZA, Robinson SM, Javaid K, Inskip HM, Godfrey KM, Dennison EM, Cooper C. Intrauterine growth and postnatal skeletal development: findings from the Southampton Women's Survey. Paediatric and perinatal epidemiology. 2012 Jan 1;26(1):34-44. |
| Heney JH, Dimock CC, Friedman JF, Lewis C. Pediatric refugees in Rhode Island: increases in BMI percentile, overweight, and obesity following resettlement. RI Med J. 2015 Jan;98(1):43-7. |
| Hernandez DC, Francis LA, Doyle EA. National School Lunch Program participation and sex differences in body mass index trajectories of children from low-income families. Archives of pediatrics & adolescent medicine. 2011 Apr 4;165(4):346-53. |
| Howe LD, Tilling K, Galobardes B, Smith GD, Gunnell D, Lawlor DA. Socioeconomic differences in childhood growth trajectories: at what age do height inequalities emerge?. Journal of Epidemiology and Community Health. 2012 Feb 1;66(2):143-8. |
| Imai CM, Gunnarsdottir I, Thorisdottir B, Halldorsson TI, Thorsdottir I. Associations between infant feeding practice prior to six months and body mass index at six years of age. Nutrients. 2014 Apr 17;6(4):1608-17. |
| Jacquot A, Neveu D, Aujoulat F, Mercier G, Marchandin H, Jumas-Bilak E, Picaud JC. Dynamics and clinical evolution of bacterial gut microflora in extremely premature patients. The Journal of pediatrics. 2011 Mar 31;158(3):390-6. |
| Johnson DE, Guthrie D, Smyke AT, Koga SF, Fox NA, Zeanah CH, Nelson CA. Growth and associations between auxology, caregiving environment, and cognition in socially deprived Romanian children randomized to foster vs ongoing institutional care. Archives of pediatrics & adolescent medicine. 2010 Jun 7;164(6):507-16. |
| Johnson W, Wright J, Cameron N. The risk of obesity by assessing infant growth against the UK-WHO charts compared to the UK90 reference: findings from the Born in Bradford birth cohort study. BMC pediatrics. 2012 Jul 23;12(1):1. |
| Johnston WH, Ashley C, Yeiser M, Harris CL, Stolz SI, Wampler JL, Wittke A, Cooper TR. Growth and tolerance of formula with lactoferrin in infants through one year of age: double-blind, randomized, controlled trial. BMC pediatrics. 2015 Nov 7;15(1):1. |
| Joseph SA, Casapía M, Montresor A, Rahme E, Ward BJ, Marquis GS, Pezo L, Blouin B, Maheu-Giroux M, Gyorkos TW. The effect of deworming on growth in one-year-old children living in a soil-transmitted helminth-endemic area of Peru: a randomized controlled trial. PLoS Negl Trop Dis. 2015 Oct 1;9(10):e0004020. |
| Jourdan C, Brugel D, Hubeaux K, Toure H, LAURENT‐VANNIER AN, Chevignard M. Weight gain after childhood traumatic brain injury: a matter of concern. Developmental Medicine & Child Neurology. 2012 Jul 1;54(7):624-8. |
| Khan AI, Hawkesworth S, Ekström EC, Arifeen S, Moore SE, Frongillo EA, Yunus M, Persson LÅ, Kabir I. Effects of exclusive breastfeeding intervention on child growth and body composition: the MINIMat trial, Bangladesh. Acta Paediatrica. 2013 Aug 1;102(8):815-23. |
| Kinlin LM, Freedman SB. Evaluation of a clinical dehydration scale in children requiring intravenous rehydration. Pediatrics. 2012 May 1;129(5):e1211-9. |
| Kong KL, Feda DM, Eiden RD, Epstein LH. Origins of food reinforcement in infants. The American journal of clinical nutrition. 2015 Mar 1;101(3):515-22. |
| Lauhkonen E, Koponen P, Nuolivirta K, Paassilta M, Toikka J, Saari A, Korppi M. Obesity and bronchial obstruction in impulse oscillometry at age 5–7 years in a prospective post‐bronchiolitis cohort. Pediatric pulmonology. 2015 Sep 1;50(9):908-14. |
| Lima PA, de Carvalho M, da Costa AC, Moreira ME. Variables associated with extra uterine growth restriction in very low birth weight infants. Jornal de pediatria. 2014 Feb 28;90(1):22-7. |
| Lin Z, Green RS, Chen S, Wu H, Liu T, Li J, Wei J, Lin J. Quantification of EUGR as a Measure of the Quality of Nutritional Care of Premature Infants. PloS one. 2015 Jul 20;10(7):e0132584. |
| Lin X, Aris IM, Tint MT, Soh SE, Godfrey KM, Yeo GS, Kwek K, Chan JK, Gluckman PD, Chong YS, Yap F. Ethnic differences in effects of maternal pre-pregnancy and pregnancy adiposity on offspring size and adiposity. The Journal of Clinical Endocrinology & Metabolism. 2015 Jul 22;100(10):3641-50. |
| Lindberg J, Norman M, Westrup B, Öhrman T, Domellöf M, Berglund SK. Overweight, obesity, and body composition in 3.5-and 7-year-old Swedish Children born with marginally low birth weight. The Journal of pediatrics. 2015 Dec 31;167(6):1246-52. |
| Linglart A, Cabrol S, Berlier P, Stuckens C, Wagner KD, De Kerdanet M, Limoni C, Carel JC, Chaussain JL, French Collaborative Young Turner Study Group. Growth hormone treatment before the age of 4 years prevents short stature in young girls with Turner syndrome. European Journal of Endocrinology. 2011 Jun 1;164(6):891-7. |
| Loomis T, Byham-Gray L, Ziegler J, Parrott JS. Impact of standardized feeding guidelines on enteral nutrition administration, growth outcomes, metabolic bone disease, and cholestasis in the NICU. Journal of Pediatric Gastroenterology and nutrition. 2014 Jul 1;59(1):93-8. |
| Lundeen EA, Stein AD, Adair LS, Behrman JR, Bhargava SK, Dearden KA, Gigante D, Norris SA, Richter LM, Fall CH, Martorell R. Height-for-age z scores increase despite increasing height deficits among children in 5 developing countries. The American journal of clinical nutrition. 2014 Sep 1;100(3):821-5. |
| Maas C, Mitt S, Full A, Arand J, Bernhard W, Poets CF, Franz AR. A historic cohort study on accelerated advancement of enteral feeding volumes in very premature infants. Neonatology. 2012 Oct 24;103(1):67-73. |
| Magnus MC, Olsen SF, Granström C, Joner G, Skrivarhaug T, Svensson J, Johannesen J, Njølstad P, Magnus P, Størdal K, Stene LC. Infant growth and risk of childhood-onset type 1 diabetes in children from 2 Scandinavian birth cohorts. JAMA pediatrics. 2015 Dec 1;169(12):e153759-. |
| Maleta KM, Phuka J, Alho L, Cheung YB, Dewey KG, Ashorn U, Phiri N, Phiri TE, Vosti SA, Zeilani M, Kumwenda C. Provision of 10–40 g/d lipid-based nutrient supplements from 6 to 18 months of age does not prevent linear growth faltering in Malawi. The Journal of nutrition. 2015 Aug 1;145(8):1909-15. |
| Martins C, Belsky J, Marques S, Baptista J, Silva J, Mesquita AR, de Castro F, Sousa N, Soares I. Diverse physical growth trajectories in institutionalized portuguese children below age 3: relation to child, family, and institutional factors. Journal of pediatric psychology. 2012 Dec 21:jss129. |
| Matijasevich A, Howe LD, Tilling K, Santos IS, Barros AJ, Lawlor DA. Maternal education inequalities in height growth rates in early childhood: 2004 Pelotas birth cohort study. Paediatric and perinatal epidemiology. 2012 May 1;26(3):236-49. |
| Mazaki-Tovi S, Kanety H, Pariente C, Hemi R, Kuint J, Yinon Y, Schiff E, Sivan E. Cord blood adiponectin and infant growth at one year. Journal of Pediatric Endocrinology and Metabolism. 2011 Aug 1;24(7-8):411-8. |
| McCloskey K, Burgner D, Carlin JB, Skilton MR, Cheung M, Dwyer T, Vuillermin P, Ponsonby AL. Infant adiposity at birth and early postnatal weight gain predict increased aortic intima-media thickness at 6 weeks of age: a population-derived cohort study. Clinical Science. 2016 Mar 1;130(6):443-50. |
| McLeod G, Sherriff J, Hartmann PE, Nathan E, Geddes D, Simmer K. Comparing different methods of human breast milk fortification using measured v. assumed macronutrient composition to target reference growth: a randomised controlled trial. British Journal of Nutrition. 2016 Feb 14;115(03):431-9. |
| Minich LL, Atz AM, Colan SD, Sleeper LA, Mital S, Jaggers J, Margossian R, Prakash A, Li JS, Cohen MS, Lacro RV. Partial and transitional atrioventricular septal defect outcomes. The Annals of thoracic surgery. 2010 Feb 28;89(2):530-6. |
| Mitter SS, Oriá RB, Kvalsund MP, Pamplona P, Joventino ES, Mota R, Gonçalves DC, Patrick PD, Guerrant RL, Lima AA. Apolipoprotein E4 influences growth and cognitive responses to micronutrient supplementation in shantytown children from northeast Brazil. Clinics. 2012;67(1):11-8. |
| Monse B, Duijster D, Sheiham A, Grijalva-Eternod CS, van Palenstein Helderman W, Hobdell MH. The effects of extraction of pulpally involved primary teeth on weight, height and BMI in underweight Filipino children. A cluster randomized clinical trial. BMC public health. 2012 Aug 31;12(1):1. |
| Nackers F, Broillet F, Oumarou D, Djibo A, Gaboulaud V, Guerin PJ, Rusch B, Grais RF, Captier V. Effectiveness of ready-to-use therapeutic food compared to a corn/soy-blend-based pre-mix for the treatment of childhood moderate acute malnutrition in Niger. Journal of tropical pediatrics. 2010 Dec 1;56(6):407-13. |
| Natarajan G, Johnson YR, Brozanski B, Farrow KN, Zaniletti I, Padula MA, Asselin JM, Durand DJ, Short BL, Pallotto EK, Dykes FD. Postnatal weight gain in preterm infants with severe bronchopulmonary dysplasia. American journal of perinatology. 2014 Mar;31(03):223-30. |
| Navardauskaite R, Dusatkova P, Obermannova B, Pfaeffle RW, Blum WF, Adukauskiene D, Smetanina N, Cinek O, Verkauskiene R, Lebl J. High prevalence of PROP1 defects in Lithuania: phenotypic findings in an ethnically homogenous cohort of patients with multiple pituitary hormone deficiency. The Journal of Clinical Endocrinology & Metabolism. 2013 Oct 31;99(1):299-306. |
| Parra-Medina D, Mojica C, Liang Y, Ouyang Y, Ramos AI, Gomez I. Promoting weight maintenance among overweight and obese Hispanic children in a rural practice. Childhood Obesity. 2015 Aug 1;11(4):355-63. |
| Peiler A, Woelfle J, Stutte S, Schreiner F, Bartmann P, Gohlke B. Postnatal nutrition in extremely low birth weight infants and its impact on growth until the age of 6 years. Acta Paediatrica. 2014 Feb 1;103(2):e61-8. |
| Perng W, Rifas-Shiman SL, Kramer MS, Haugaard LK, Oken E, Gillman MW, Belfort MB. Early weight gain, linear growth, and mid-childhood blood pressure a prospective study in project viva. Hypertension. 2016 Feb 1;67(2):301-8. |
| Pimpin L, Jebb S, Johnson L, Wardle J, Ambrosini GL. Dietary protein intake is associated with body mass index and weight up to 5 y of age in a prospective cohort of twins. The American journal of clinical nutrition. 2016 Feb 1;103(2):389-97. |
| Radhakrishna KV, Kulkarni B, Balakrishna N, Rajkumar H, Omkar C, Shatrugna V. Composition of weight gain during nutrition rehabilitation of severely under nourished children in a hospital based study from India. Asia Pacific journal of clinical nutrition. 2010 Mar 1;19(1):8-13. |
| Kumar TR, Ramji S. Effect of zinc supplementation on growth in very low birth weight infants. Journal of tropical pediatrics. 2012 Feb 1;58(1):50-4. |
| Ramel SE, Gray HL, Ode KL, Younge N, Georgieff MK, Demerath EW. Body composition changes in preterm infants following hospital discharge: comparison with term infants. Journal of pediatric gastroenterology and nutrition. 2011 Sep 1;53(3):333-8. |
| Ranke MB, Schweizer R, Martin DD, Ehehalt S, Schwarze CP, Serra F, Binder G. Analyses from a centre of short-and long-term growth in Turner’s syndrome on standard growth hormone doses confirm growth prediction algorithms and show normal IGF-I levels. Hormone Research in Paediatrics. 2012 Mar 16;77(4):214-21. (Ranke 2012a) |
| Ranke MB, Lindberg A, Brosz M, Kaspers S, Loftus J, Wollmann H, Kołtowska-Haggstrom M, Roelants M. Accurate long-term prediction of height during the first four years of growth hormone treatment in prepubertal children with growth hormone deficiency or Turner Syndrome. Hormone research in pædiatrics. 2012 Jul 23;78(1):8-17. (Ranke 2012b) |
| Reeske A, Spallek J, Bammann K, Eiben G, De Henauw S, Kourides Y, Nagy P, Ahrens W. Migrant background and weight gain in early infancy: results from the German study sample of the IDEFICS study. PloS one. 2013 Apr 4;8(4):e60648. |
| Rogers SL, Hughes BA, Jones CA, Freedman L, Smart K, Taylor N, Stewart PM, Shackleton CH, Krone NP, Blissett J, Tomlinson JW. Diminished 11β-hydroxysteroid dehydrogenase type 2 activity is associated with decreased weight and weight gain across the first year of life. The Journal of Clinical Endocrinology & Metabolism. 2014 Feb 11;99(5):E821-31. |
| Saari A, Harju S, Mäkitie O, Saha MT, Dunkel L, Sankilampi U. Systematic growth monitoring for the early detection of celiac disease in children. JAMA pediatrics. 2015 Mar 1;169(3):e1525-. |
| Saleem AF, Mahmud S, Baig-Ansari N, Zaidi AK. Impact of maternal education about complementary feeding on their infants' nutritional outcomes in low-and middle-income households: a community-based randomized interventional study in Karachi, Pakistan. Journal of health, population, and nutrition. 2014 Dec;32(4):623. |
| Sanders DB, Fink A, Mayer-Hamblett N, Schechter MS, Sawicki GS, Rosenfeld M, Flume PA, Morgan WJ. Early life growth trajectories in cystic fibrosis are associated with pulmonary function at age 6 years. The Journal of pediatrics. 2015 Nov 30;167(5):1081-8. |
| Schreiner F, Gohlke B, Stutte S, Bartmann P, Woelfle J. Growth hormone receptor d3-variant, insulin-like growth factor binding protein-1–575G/A polymorphism and postnatal catch-up growth: Association with parameters of glucose homeostasis in former extremely low birth weight preterm infants. Growth Hormone & IGF Research. 2010 Jun 30;20(3):201-4. |
| Silventoinen K, Pitkäniemi J, Latvala A, Kaprio J, Yokoyama Y. Association between physical and motor development in childhood: a longitudinal study of Japanese twins. Twin research and human genetics. 2014 Jun 1;17(03):192-8. |
| Skoner DP, Meltzer EO, Milgrom H, Stryszak P, Teper A, Staudinger H. Effects of inhaled mometasone furoate on growth velocity and adrenal function: a placebo-controlled trial in children 4–9 years old with mild persistent asthma. Journal of Asthma. 2011 Oct 1;48(8):848-59. |
| Spiegler J, Preuß M, Gebauer C, Bendiks M, Herting E, Göpel W, German Neonatal Network. Does breastmilk influence the development of bronchopulmonary dysplasia?. The Journal of pediatrics. 2016 Feb 29;169:76-80. |
| Stark LJ, Clifford LM, Towner EK, Filigno SS, Zion C, Bolling C, Rausch J. A pilot randomized controlled trial of a behavioral family-based intervention with and without home visits to decrease obesity in preschoolers. Journal of pediatric psychology. 2014 Oct 1;39(9):1001-12. |
| Surkan PJ, Ettinger AK, Hock RS, Ahmed S, Strobino DM, Minkovitz CS. Early maternal depressive symptoms and child growth trajectories: a longitudinal analysis of a nationally representative US birth cohort. BMC pediatrics. 2014 Jul 21;14(1):1. |
| Suzuki K, Kondo N, Sato M, Tanaka T, Ando D, Yamagata Z. Gender differences in the association between maternal smoking during pregnancy and childhood growth trajectories: multilevel analysis. International Journal of Obesity. 2011 Jan 1;35(1):53-9. |
| Tandon N, Fall CH, Osmond C, Sachdev HP, Prabhakaran D, Ramakrishnan L, Biswas SD, Ramji S, Khalil A, Gera T, Reddy KS. Growth from birth to adulthood and peak bone mass and density data from the New Delhi Birth Cohort. Osteoporosis International. 2012 Oct 1;23(10):2447-59. |
| Taylor M, Couto-Silva AC, Adan L, Trivin C, Sainte-Rose C, Zerah M, Valteau-Couanet D, Doz F, Chalumeau M, Brauner R. Hypothalamic-pituitary lesions in pediatric patients: endocrine symptoms often precede neuro-ophthalmic presenting symptoms. The Journal of pediatrics. 2012 Nov 30;161(5):855-63. |
| Thakwalakwa C, Ashorn P, Phuka J, Cheung YB, Briend A, Puumalainen T, Maleta K. A lipid-based nutrient supplement but not corn-soy blend modestly increases weight gain among 6-to 18-month-old moderately underweight children in rural Malawi. The Journal of nutrition. 2010 Nov 1;140(11):2008-13. |
| Thakwalakwa CM, Ashorn P, Jawati M, Phuka JC, Cheung YB, Maleta KM. An effectiveness trial showed lipid-based nutrient supplementation but not corn–soya blend offered a modest benefit in weight gain among 6-to 18-month-old underweight children in rural Malawi. Public health nutrition. 2012 Sep 1;15(09):1755-62. |
| Thearle MS, Votruba SB, Piaggi P, Muller YL, Hanson RL, Baier LJ, Knowler W, Krakoff J. The effect of differing patterns of childhood body mass index gain on adult physiology in American Indians. Obesity. 2015 Sep 1;23(9):1872-80. |
| Thomas N, Cherian A, Santhanam S, Jana AK. A randomized control trial comparing two enteral feeding volumes in very low birth weight babies. Journal of tropical pediatrics. 2012 Feb 1;58(1):55-8. |
| Trehan I, Goldbach HS, LaGrone LN, Meuli GJ, Wang RJ, Maleta KM, Manary MJ. Antibiotics as part of the management of severe acute malnutrition. New England Journal of Medicine. 2013 Jan 31;368(5):425-35. |
| Tu AW, Mâsse LC, Lear SA, Gotay CC, Richardson CG. Body mass index trajectories from ages 1 to 20: Results from two nationally representative canadian longitudinal cohorts. Obesity. 2015 Aug 1;23(8):1703-11. |
| Vieira SA, Magalhães TC, Ribeiro AQ, Priore SE, Franceschini SD, Sant’Ana LF. Influence of weight gain rate on early life nutritional status and body composition of children. The Scientific World Journal. 2014 Nov 4;2014. |
| Wasniewska M, Aversa T, Mazzanti L, Guarneri MP, Matarazzo P, De Luca F, Lombardo F, Messina MF, Valenzise M. Adult height in girls with Turner syndrome treated from before 6 years of age with a fixed per kilogram GH dose. European Journal of Endocrinology. 2013 Oct 1;169(4):439-43. |
| Wijlaars LP, Johnson L, van Jaarsveld CH, Wardle J. Socioeconomic status and weight gain in early infancy. International journal of obesity. 2011 Jul 1;35(7):963-70. |
| Wright CM, Chillingworth A. The impact of stopping high-energy oral nutritional supplements on eating behaviour and weight gain. Archives of disease in childhood. 2015 Mar 25:archdischild-2014. |
| Yanagida N, Minoura T, Kitaoka S. Does Terminating the Avoidance of Cow's Milk Lead to Growth in Height?. International archives of allergy and immunology. 2015 Nov 12;168(1):56-60. |
| Yang Q, Ayers K, Welch CD, O'Shea TM. Randomized controlled trial of early enteral fat supplement and fish oil to promote intestinal adaptation in premature infants with an enterostomy. The Journal of pediatrics. 2014 Aug 31;165(2):274-9. |
| Zhao Z, Ding M, Hu Z, Dai Q, Satija A, Zhou A, Xu Y, Zhang X, Hu FB, Xu H. Trajectories of length, weight, and bone mineral density among preterm infants during the first 12 months of corrected age in China. BMC pediatrics. 2015 Aug 5;15(1):1. |
| Zheng M, Rangan A, Allman-Farinelli M, Rohde JF, Olsen NJ, Heitmann BL. Replacing sugary drinks with milk is inversely associated with weight gain among young obesity-predisposed children. British Journal of Nutrition. 2015 Nov 14;114(09):1448-55. |
| Zung A, Kori M. Lack of association between seroconversion and catch-up growth in children with celiac disease. Journal of Pediatric Endocrinology and Metabolism. 2012 Feb 1;25(1-2):131-7. |
| de Hoog ML, van Eijsden M, Stronks K, Gemke RJ, Vrijkotte TG. Overweight at age two years in a multi-ethnic cohort (ABCD study): the role of prenatal factors, birth outcomes and postnatal factors. BMC public health. 2011 Aug 1;11(1):1. |
| de Ruyter JC, Olthof MR, Seidell JC, Katan MB. A trial of sugar-free or sugar-sweetened beverages and body weight in children. New England Journal of Medicine. 2012 Oct 11;367(15):1397-406. |
| ud Din Z, Emmett P, Steer C, Emond A. Growth outcomes of weight faltering in infancy in ALSPAC. Pediatrics. 2013 Mar 1;131(3):e843-9. |
| Van Gool SA, Kamp GA, Odink RJ, de Muinck Keizer-Schrama SM, Delemarre-van de Waal HA, Oostdijk W, Wit JM. High-dose GH treatment limited to the prepubertal period in young children with idiopathic short stature does not increase adult height. European Journal of Endocrinology. 2010 Apr 1;162(4):653-60. |
| van der Gugten AC, Koopman M, Evelein AM, Verheij TJ, Uiterwaal CS, van der Ent CK. Rapid early weight gain is associated with wheeze and reduced lung function in childhood. European Respiratory Journal. 2012 Feb 1;39(2):403-10. |
| Van der Willik EM, Vrijkotte TG, Altenburg TM, Gademan MG, Kist-van Holthe J. Exclusively breastfed overweight infants are at the same risk of childhood overweight as formula fed overweight infants. Archives of disease in childhood. 2015 Oct 1;100(10):932-7. |
