# Supplementary material for: Metrics of early childhood growth in recent epidemiological research: A scoping review
Source: PLoS One. 2018 Mar 20;13(3):e0194565. doi: 10.1371/journal.pone.0194565 (PMC5860780; doi:10.1371/journal.pone.0194565)
Supplement: S5 File — Content Signatures. (DOCX) [file pone.0194565.s005.docx]

# **E. Content Signatures**

## **Table E1.** Content signatures for growth in length

| Content Signatures^a^ | *n* |
| --- | --- |
| **Exposure** | |
| Total | 20 |
| 12121311 | 1 |
| 12121411 | 2 |
| 12131413 | 1 |
| 12131417 | 2 |
| 22121311 | 7 |
| 22121417 | 1 |
| 22121819 | 3 |
| 22122411 | 1 |
| 22131313 | 1 |
| 22232312 | 1 |
| **Outcome** | |
| Total | 67 |
| 11131615 | 1 |
| 12121311 | 7 |
| 12121411 | 7 |
| 12121414 | 1 |
| 12121415 | 1 |
| 12121417 | 2 |
| 12131414 | 1 |
| 12131417 | 7 |
| 12131513 | 1 |
| 12131713 | 1 |
| 21232322 | 1 |
| 22121311 | 23 |
| 22121317 | 1 |
| 22121411 | 1 |
| 22121819 | 3 |
| 22122011 | 1 |
| 22122224 | 1 |
| 22122411 | 1 |
| 22131315 | 1 |
| 22131417 | 1 |
| 22222312 | 3 |
| 22232312 | 1 |

^a^ The range of content signatures generated is based on random sample of published studies, and therefore is not exhaustive

## **Table E2.** Content signatures for growth in weight

| Content Signatures^a^ | *n* |
| --- | --- |
| **Exposure** | |
| Total | 24 |
| 12121311 | 2 |
| 12121411 | 4 |
| 12121611 | 1 |
| 12131417 | 2 |
| 12222312 | 3 |
| 22121311 | 6 |
| 22121417 | 1 |
| 22121819 | 1 |
| 22131313 | 1 |
| 22222312 | 2 |
| 22232312 | 1 |
| **Outcome** | |
| Total | 75 |
| 11131615 | 1 |
| 12121311 | 15 |
| 12121411 | 5 |
| 12121414 | 1 |
| 12121415 | 1 |
| 12121417 | 1 |
| 12121611 | 3 |
| 12121711 | 6 |
| 12121717 | 1 |
| 12131417 | 3 |
| 12131418 | 1 |
| 12131513 | 1 |
| 12131617 | 1 |
| 12131713 | 1 |
| 12132312 | 1 |
| 12222312 | 1 |
| 21232322 | 1 |
| 22121311 | 23 |
| 22121317 | 1 |
| 22121411 | 1 |
| 22121819 | 1 |
| 22122411 | 1 |
| 22131315 | 1 |
| 22131417 | 1 |
| 22222312 | 2 |

^a^ The range of content signatures generated is based on random sample of published studies, and therefore is not exhaustive

## **Table E3.** Content signatures for growth in BMI

| Content Signatures | *n* |
| --- | --- |
| **Exposure** | |
| Total | 11 |
| 11232322 | 1 |
| 12131417 | 1 |
| 21232322 | 2 |
| 22121311 | 2 |
| 22121417 | 1 |
| 22121819 | 1 |
| 22122224 | 1 |
| 22131313 | 1 |
| 22232312 | 1 |
| **Outcome** | |
| Total | 38 |
| 11131415 | 1 |
| 11232321 | 1 |
| 11232322 | 1 |
| 12121311 | 3 |
| 12131417 | 5 |
| 21131315 | 1 |
| 21131415 | 1 |
| 21232321 | 1 |
| 21232322 | 1 |
| 22121311 | 12 |
| 22121819 | 1 |
| 22122224 | 1 |
| 22131317 | 1 |
| 22131417 | 5 |
| 22132312 | 1 |
| 22222312 | 2 |

^a^ The range of content signatures generated is based on random sample of published studies, and therefore is not exhaustive
