# Supplementary material for: Metrics of early childhood growth in recent epidemiological research: A scoping review
Source: PLoS One. 2018 Mar 20;13(3):e0194565. doi: 10.1371/journal.pone.0194565 (PMC5860780; doi:10.1371/journal.pone.0194565)
Supplement: S6 File — Label-to-content matrices. (DOCX) [file pone.0194565.s006.docx]

# **F. Label-to-Content Matrices**


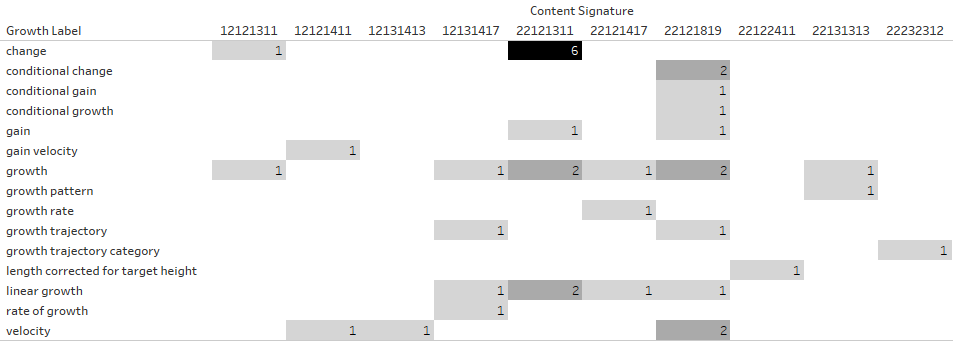


**Figure F1.** Metric 8-digit content signatures for growth in length as an exposure with author-assigned labels. Shaded cells highlight growth metric usage in at least one study, and darker shading indicates increased frequencies of usage. The range of content signatures presented is based on a random sample of published studies, and therefore is not exhaustive.


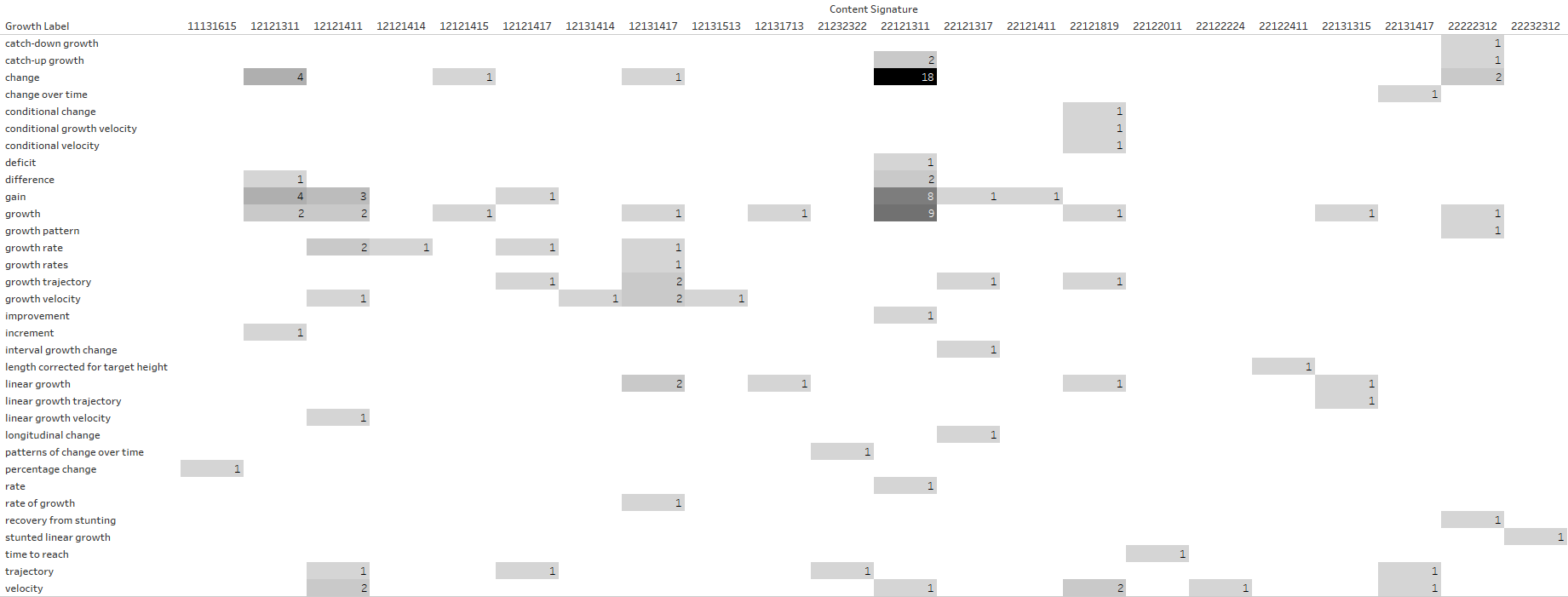


**Figure F2.** Metric 8-digit content signatures for growth in length as an outcome with author-assigned labels. Shaded cells highlight growth metric usage in at least one study, and darker shading indicates increased frequencies of usage. The range of content signatures presented is based on a random sample of published studies, and therefore is not exhaustive.


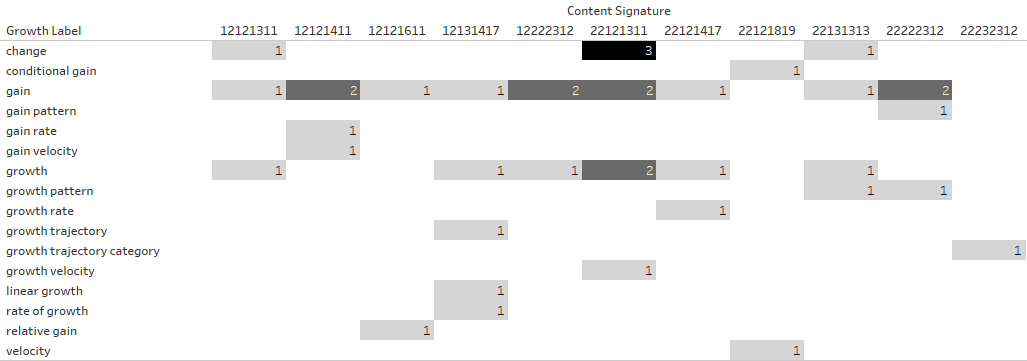


**Figure F3.** Metric 8-digit content signatures for growth in weight as an exposure with author-assigned labels. Shaded cells highlight growth metric usage in at least one study, and darker shading indicates increased frequencies of usage. The range of content signatures presented is based on a random sample of published studies, and therefore is not exhaustive.


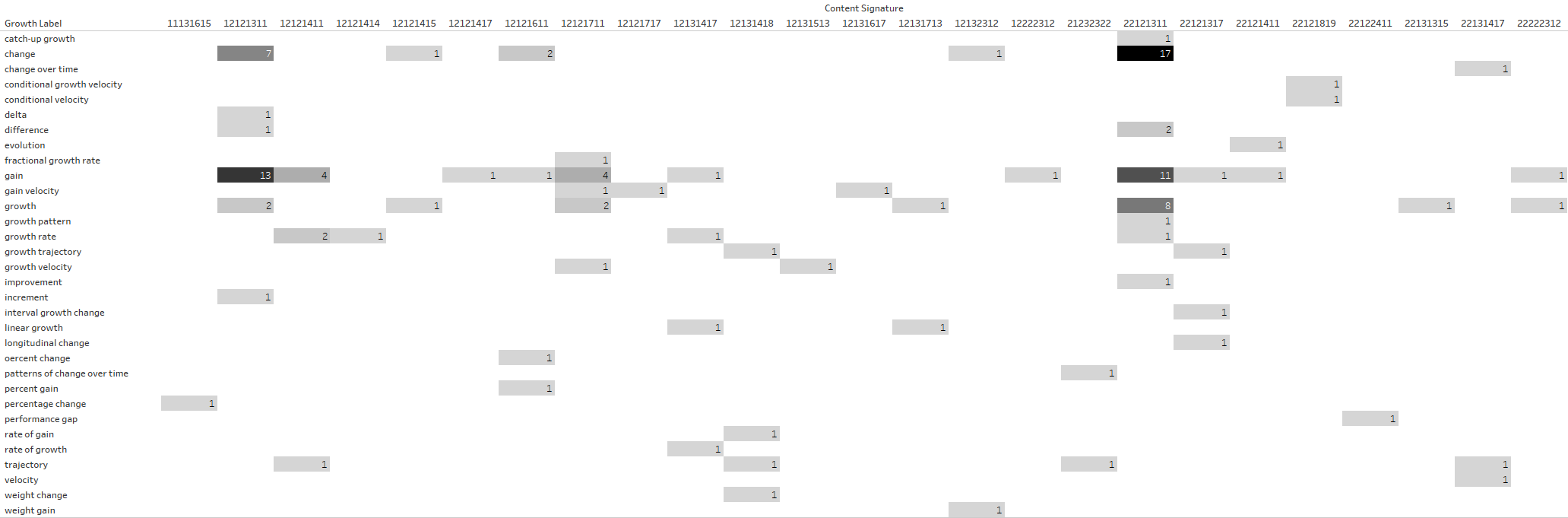


**Figure F4.** Metric 8-digit content signatures for growth in weight as an outcome with author-assigned labels. Shaded cells highlight growth metric usage in at least one study, and darker shading indicates increased frequencies of usage. The range of content signatures presented is based on a random sample of published studies, and therefore is not exhaustive.


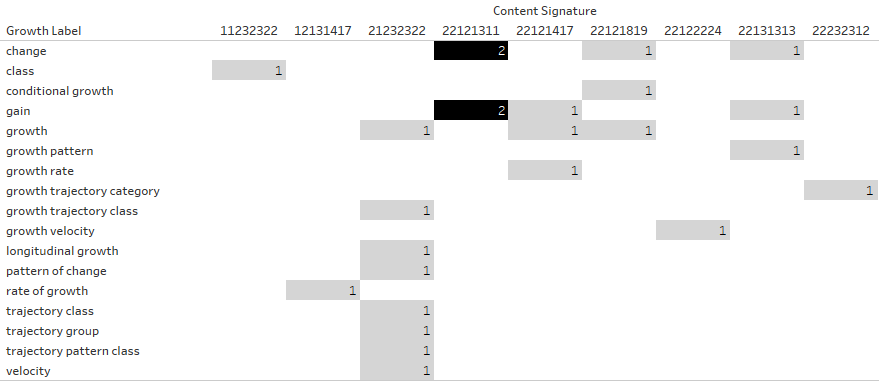


**Figure F5.** Metric 8-digit content signatures for growth in BMI as an exposure with author-assigned labels. Shaded cells highlight growth metric usage in at least one study, and darker shading indicates increased frequencies of usage. The range of content signatures presented is based on a random sample of published studies, and therefore is not exhaustive.


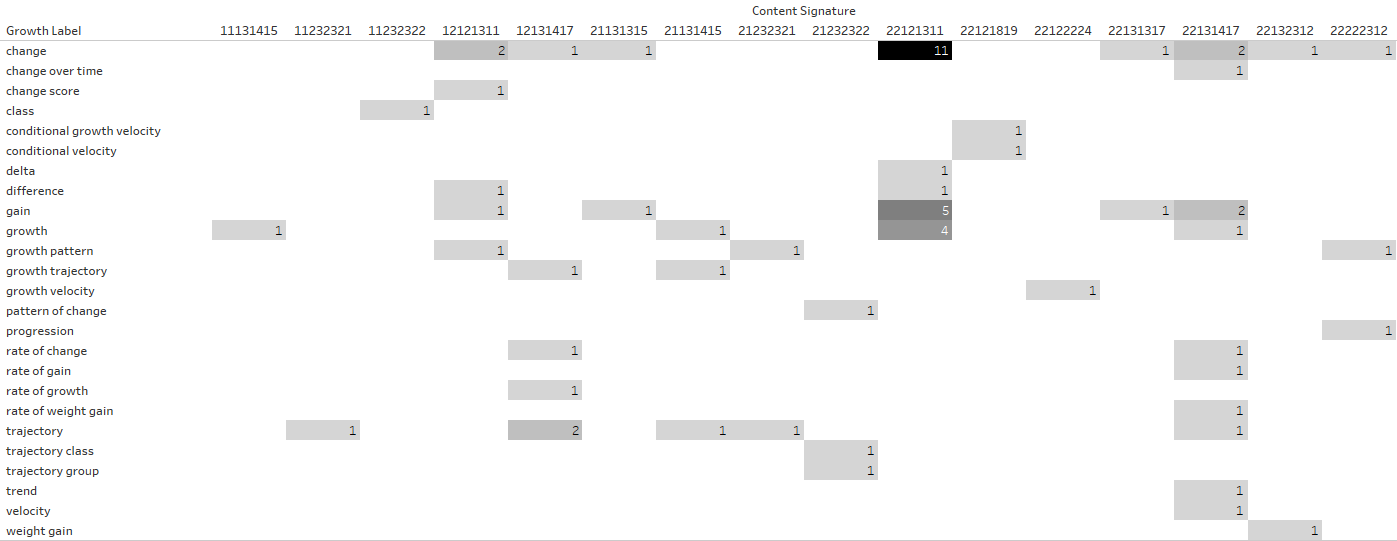


**Figure F6.** Metric 8-digit content signatures for growth in BMI as an outcome with author-assigned labels. Shaded cells highlight growth metric usage in at least one study, and darker shading indicates increased frequencies of usage. The range of content signatures presented is based on a random sample of published studies, and therefore is not exhaustive.
